# Novel role for caspase 1 inhibitor VX765 in suppressing NLRP3 inflammasome assembly and atherosclerosis via promoting mitophagy and efferocytosis

**DOI:** 10.1038/s41419-022-04966-8

**Published:** 2022-05-31

**Authors:** Ying Jin, Yao Liu, Lei Xu, Jie Xu, Yulian Xiong, Yazhi Peng, Ke Ding, Shuang Zheng, Nan Yang, Zemei Zhang, Lin Li, Liguo Tan, HongXian Song, Jian Fu

**Affiliations:** 1 The Laboratory of Inflammation and Vascular Biology, Institute of Clinical Medicine and Department of Cardiology, Renmin Hospital, Hubei University of Medicine, Shiyan, Hubei China; 2 Department of Ultrasound, Renmin Hospital, Hubei University of Medicine, Shiyan, Hubei China; 3 Graduate School, Jinzhou Medical University, Jinzhou, Liaoning China; 4 Department of Cardiology, Renmin Hospital, Hubei University of Medicine, Shiyan, Hubei China

**Keywords:** Mitophagy, Atherosclerosis

## Abstract

Atherosclerosis is a maladaptive chronic inflammatory disease, which remains the leading cause of death worldwide. The NLRP3 inflammasome constitutes a major driver of atherosclerosis, yet the mechanism of action is poorly understood. Mitochondrial dysfunction is essential for NLRP3 inflammasome activation. However, whether activated NLRP3 inflammasome exacerbates mitochondrial dysfunction remains to be further elucidated. Herein, we sought to address these issues applying VX765, a well-established inhibitor of caspase 1. VX765 robustly restrains caspase 1-mediated interleukin-1β production and gasdermin D processing. Our study assigned VX765 a novel role in antagonizing NLRP3 inflammasome assembly and activation. VX765 mitigates mitochondrial damage induced by activated NLRP3 inflammasome, as evidenced by decreased mitochondrial ROS production and cytosolic release of mitochondrial DNA. VX765 blunts caspase 1-dependent cleavage and promotes mitochondrial recruitment and phosphorylation of Parkin, a key mitophagy regulator. Functionally, VX765 facilitates mitophagy, efferocytosis and M2 polarization of macrophages. It also impedes foam cell formation, migration and pyroptosis of macrophages. VX765 boosts autophagy, promotes efferocytosis, and alleviates vascular inflammation and atherosclerosis in both *ApoE*^−/−^ and *Ldlr*^−/−^ mice. However, these effects of VX765 were abrogated upon ablation of *Nlrp3* in *ApoE*^−/−^ mice. This work provides mechanistic insights into NLRP3 inflammasome assembly and this inflammasome in dictating atherosclerosis. This study highlights that manipulation of caspase 1 paves a new avenue to treatment of atherosclerotic cardiovascular disease.

## Introduction

Atherosclerosis has been appreciated as a maladaptive chronic inflammatory disorder that is ignited by subendothelial buildup of low-density lipoproteins (LDL) [1]. Remarkably, the Canakinumab Antiinflammatory Thrombosis Outcome Study trial furnishes definitive evidence that inhibition of interleukin (IL)-1β with its monoclonal antibody canakinumab robustly reduced cardiovascular events for patients with atherosclerotic cardiovascular disease (ASCVD) [2], allowing the targeting inflammation to clinical realty.

IL-1β is synthesized in cells (primarily macrophages) as a biologically inactive precursor (termed pro-IL-1β). Upon the activation of the nucleotide-binding oligomerization domain, leucine-rich repeat-containing receptor family pyrin domain-containing 3 (NLRP3) inflammasome, pro-IL-1β is processed by activated caspase 1 to form active IL-1β [3]. The NLRP3 inflammasome is a protein complex composed of NLRP3, apoptosis-associated speck-like protein containing a CARD (ASC), and procaspase 1 [3]. Upon NLRP3 inflammasome activation, activated caspase 1 mediates proteolytic cleavage of different protein substrates, notably pro-IL-1β and gasdermin D (GSDMD), culminating in pyroptosis [3]. NLRP3 inflammasome assembly and activation requires two signals: priming signal and activation signal [3]. Activation signal initiates NLRP3 inflammasome assembly through igniting various mechanisms involving mitochondrial dysfunction [4, 5]. It is well appreciated that mitochondrial damage is a trigger of NLRP3 inflammasome activation in response to the NLRP3 activators [6, 7]. However, whether NLRP3 inflammasome activation triggers/aggravates mitochondrial damage is unclear. Clarifying this issue will greatly improve our understanding of the mechanism behind NLRP3 inflammasome assembly and activation.

The fact that the NLRP3 inflammasome plays the causal role in driving atherosclerosis is transforming the field of atherosclerosis [3]. Nonetheless, several critical questions remain to be addressed. The mechanisms whereby the NLRP3 inflammasome orchestrates atherosclerosis remain to be completely elucidated [3]. NLRP3 inflammasome activation leads to production of active IL-1β and IL-18 as well as pyroptosis, all of which play profound roles in atherosclerosis. However, virtually nothing is known regarding whether the NLRP3 inflammasome dictates vascular inflammation and atherosclerosis through regulating autophagy and efferocytosis, a process by which apoptotic cells in tissues are engulfed by phagocytes [8]. Additionally, there is a long-standing puzzle of why genetic studies yielded mixed results. In short, *LDL receptor* (*Ldlr*)–deficient (*Ldlr*^−/−^) mice transplanted with bone marrow lacking *Nlrp3*, *Asc*, or *IL-1β* were athero-protective [9]. In sharp contrast, a deletion of the NLRP3 inflammasome component in *Apoprotein E*–deficient (*ApoE*^*−/−*^) mice had no significant impact on atherosclerosis [10].

These issues spur us to test the role and mechanism for caspase 1 inhibition in regulating vascular inflammation and atherosclerosis. Our study demonstrated that VX765, a well-recognized selective caspase 1 inhibitor [11], restricts activation and activity of caspase 1. Critically important is that our study assigns VX765 a novel function in antagonizing NLRP3 inflammasome assembly and activation. VX765 alleviates mitochondrial damage and promotes mitophagy. This work reveals the functions of VX765 in regulating properties of macrophages associated with atherosclerosis. We demonstrated that VX765 ameliorates vascular inflammation and atherosclerosis in both *Ldlr*^*−/−*^ and *ApoE*^*−/−*^ models. Our study provides mechanistic insights into the NLRP3 inflammasome in vascular inflammation and atherosclerosis.

## Materials and methods

### Cell culture, plasmids and transfection

Human embryonic kidney 293 T (HEK293T) cells, Jurkat T cells, mouse RAW264.7 macrophages, J774A.1 macrophages, and L929 cells were purchased from ATCC (Manassas, VA). The cells were maintained in Dulbecco’s modified Eagle’s medium (DMEM) or RPMI 1640 (for Jurkat cells) supplemented with 10% heat-inactivated fetal bovine serum (FBS). All cell lines were verified to be mycoplasma-free. HEK293T cells were transfected with Lipofectamine 2000 reagent (Invitrogen) as described previously [12]. Plasmids expressing Flag-NLRP3, hemagglutinin (HA)-NEK7 (never in mitosis gene a-related kinase 7), Flag-ASC, Flag-procaspase 1 and Flag-pro-IL-1β were described previously [13]. pEGFP-*parkin* was from Prof. L. Wang (Wuhan University, Wuhan, China) [14].

### Preparation of bone marrow-derived macrophages (BMDMs)

Mouse BMDMs were prepared by flushing the bone marrow from the tibiae and femorae as described previously [13]. Cells were differentiated into macrophages by culturing in DMEM supplemented with 10% FBS plus 20% L929-conditioned media for 7 days. To induce the activation of the NLRP3 inflammasome, macrophages were first primed with lipopolysaccharides (LPS; 100 ng/mL, 8 h) prior to stimulation with ATP (5 mM, 1 h) for different times as indicated.

### Quantitative reverse transcription-PCR (RT-qPCR)

Total RNA was extracted from the cells using Trizol reagent (Invitrogen). Reverse transcription (RT) of mRNA was performed using a RevertAid First Strand cDNA Synthesis Kit (Thermo Fisher Scientific). qPCR was conducted using a FastStart Universal SYBR Green Master (Roche Applied Science) and run on a Real‐time PCR System (ABI‐7000). Following the amplification, the Ct values for target genes and the reference gene, *GAPDH*, were recorded. Fold induction was calculated using the ΔΔCt method. Primer sequences are available upon request.

### Immunoblotting

Immunoblotting was conducted as described previously [15, 16]. Primary antibodies applied in this study include the following: anti-NLRP3 (AdipoGen, AG-20B-0014-C100), anti-ASC (AdipoGen, AG-25B-0006), anti-caspase 1 (AdipoGen, AG-20B-0042-C100), anti-interleukin-1β (R&D Systems, AF-401-NA), anti-FLAG (Sigma, F1804), anti-HA (Biolegend, 901501), anti-myc (Santa Cruz Biotechnology, sc-40), anti-gasdermin D (Abcam, ab209845), anti-ABCA1 (Abcam, ab18180), anti-CD36 (Novus Biologicals, NB400-144), anti-Parkin (Cell Signaling Technology, #4211), anti-phosphorylated Parkin Serine 65 (Affinity, AF3500), anti-VDAC1 (Cell Signaling Technology, #4661), anti-LC3 (Sigma, L8918), anti-p62 (MBL, PM045), anti-caspase 8 (Novus Biologicals, NB100-56116), anti-cleaved caspase 3 (Cell Signaling Technology, #9661), anti-arginase 1 (Cell Signaling Technology, #9819), anti-PARP (Cell Signaling Technology, #9542), anti-α-tubulin (Antgene, Wuhan, China; ANT328), anti-β-actin (Yeasen, Shanghai, China; 30101ES50), and anti-GAPDH (Antgene, Wuhan, China; ANT324).

### Immunoprecipitation

Immunoprecipitation was performed as previously described [12, 17]. Cells were solubilized in lysis buffer. The precleared lysates were incubated with the corresponding antibody (about 1–1.5 μg each) in the presence of 20 μl of Protein A/G Agarose (Pierce) overnight with constant agitation. After extensive washing, the immunoprecipitates were subjected to immunoblotting.

### Detection of proteins in cultured cell medium

The cultured cell media were concentrated by chloroform and methanol precipitation as described previously [13]. In brief, the cultured media collected and mixed with 1/4 volume (V) of chloroform plus 1 V of methanol. The samples were centrifuged at 15,000 *g* for 10 min at room temperature (RT). After removing the water/methanol mix, an additional 1 V of methanol was added, followed by centrifugation at 15,000 *g* for 10 min at RT. The protein pellet was air dried for 5 min at RT, resuspended in Triton-based lysis buffer [18] and analyzed by immunoblotting.

### Reconstitution of the NLRP3 inflammasome in HEK293T cells

HEK293T cells were seeded in 24-well plates at a density of 2 × 10^5^ cells per well. The cells were transfected with the plasmids expressing Flag-NLRP3 (200 ng), HA-NEK7 (200 ng), Flag- or myc-ASC (20 ng), Flag-procaspase1 (100 ng) and Flag-pro-IL-1β (200 ng). The cultured media were then changed at 36 h post-transfection and the cells cultured for an additional 12 h. The concentrated media and cell lysates were examined by immunoblotting.

### Semi-denaturing detergent agarose gel electrophoresis (SDD-AGE)

To assess NLRP3 oligomerization, SDD-AGE was carried out as previously described [13] with minor modifications. Cells were harvested in lysis buffer for 40 min at 4 °C. The cleared lysates were resuspended in buffer containing 0.5 × Tris borate EDTA, 2% SDS, 10% glycerol, and 0.0025% bromophenol blue. The samples were resolved on a 1.5% vertical agarose gel in running buffer containing 1 × Tris borate EDTA (89 mM Tris, pH 8.3, 89 mM boric acid, and 2 mM EDTA) and 0.1% SDS for 75 min with a voltage of 80 V. The proteins were blotted onto a polyvinylidenedifluoride membrane for 3 h, followed by immunoblotting with anti-NLRP3.

### ASC oligomerization assay

Macrophages were harvested in lysis buffer (50 mM Tris–HCl, pH 7.5, 150 mM NaCl, 10% glycerol, 0.5% Triton X-100, 1 mM PMSF, and complete protease inhibitor cocktail) and incubated on ice for 30 min, followed by centrifugation at 6000 *g* for 15 min at 4 °C. The supernatants and pellets were used as the Triton-soluble and –insoluble fractions, respectively. To detect ASC oligomerization, the Triton-insoluble fractions were washed with lysis buffer and the pellets were resuspended in 500 μl of lysis buffer. The pellets were cross-linked for 30 min at 37 °C with 2 mM disuccinimidyl suberate (Pierce) and then spun down for 15 min at 3300 *g*. The pellets were eluted and analyzed by immunoblotting with anti-ASC antibody.

### Macrophage polarization assay

BMDMs were plated into 35 mm culture dishes at a density of 1 × 10^6^ cells per well and polarized toward an M1 phenotype by incubation with LPS (100 ng/mL) and interferon (INFγ, 50 ng/mL; R&D Systems) or toward an M2 phenotype by exposure to interleukin-4 (20 ng/mL; R&D Systems) for 24 h. Total RNA or total protein lysates were prepared and analyzed by RT-qPCR or immunoblotting, respectively.

### In vitro macrophage migration assay

In vitro migration of macrophages was assessed using a wound-healing assay. In brief, 1 × 10^6^ macrophages were seeded in 35 mm culture dishes and incubated in the complete medium overnight. The confluent monolayer of the cells was scratched straightly with a 100 μl pipette tip, and the cellular debris rinsed away with PBS. Migration was visualized under a microscope and the images acquired at the indicated time points. The cells in the denuded regions were counted and analyzed.

### Generating apoptotic Jurkat cells

Jurkat cells were seeded at a density of 1 × 10^6^/mL in fresh cell culture medium and labeled with Calcein AM at 37 °C for 2 h. Labeling was monitored under a fluorescence microscope. After thoroughly washing to remove unincorporated dye, cells were resuspended into fresh culture media. For induction of apoptosis, staurosporine was added to the labeled cells to a final concentration of 1 μM for 2 h.

### In vitro efferocytosis assay

In vitro efferocytosis analysis was conducted as described previously [17]. About 2.5 × 10^5^ BMDMs were seeded onto 24-well tissue culture plate and grown overnight. Around 5 × 10^5^ apoptotic Jurkat T cells were loaded onto macrophages and incubated at 37 °C for 45 min. Level of engulfment was monitored under fluorescent microscope. Apoptotic cells (ACs) were washed away with PBS. The cells were fixed with 4% paraformaldehyde for 10 min. The images were acquired and subjected to quantitation of efferocytotic cells.

### In situ efferocytosis analysis

In situ efferocytosis analysis was performed as described previously [19]. Apoptotic cells (ACs) in paraffin-embedded tissue sections were identified by detection of active caspase 3 using immunofluorescent staining with anti-cleaved caspase 3 (Cell Signaling Technology, #9661) or terminal deoxynucleotidyl transferase dUTP nick end labeling (TUNEL) using the in situ cell death detection kit (Promega). Lesional macrophages were identified with anti-Mac3 (BD Pharmingen, #550292). Images were captured using a fluorescent microscope. The number of ACs that were co-localized with or adjacent to macrophages (named associated ACs) and the number of ACs that were not associated with macrophages (called free ACs) were counted. Efferocytosis efficiency was expressed as the ratio of associated ACs:free ACs per tissue section.

### Immunohistochemistry and double immunofluorescence staining

Immunohistochemistry was conducted as described previously [13, 20]. Mouse tissues were fixed in 60% methanol and 10% acetic acid in H_2_O and paraffin embedded. Following deparaffinization and rehydration, tissue sections were treated with 3% hydrogen peroxide solution for 20 min to block endogenous peroxides activity. Antigen retrieval was carried out by treatment of the slides with 10 mM sodium citrate buffer (pH 6.0) or EDTA (pH 9.0) by microwaving for 10 min. The samples were blocked with 5% FBS in 0.1% PBS/BSA and incubated with primary antibody overnight at 4 °C. The standard streptavidin–biotin linked horseradish peroxidase technique was applied with 3,30-diaminobenzidine tetrahydrochloride for the development of peroxidase activity. The sections were counterstained with hematoxylin.

Double immunofluorescence staining was performed as described previously [13]. Primary antibodies used include the following: anti-α-smooth actin (Santa Cruz Biotechnology, sc-130616), anti-MCP1 (R&D systems, 479-JE-010; Novus Biologicals, NBP2-22115), anti-active caspase 1 (Invitrogen, AAC504N), anti-NLRP3 (AdipoGen, AG-20B-0014), anti-interleukin-1β (R&D systems, AF-401-NA), anti-LC3 (Sigma, L8918), anti-p62 (MBL, PM045), anti-Mac3 (BD Pharmingen, #550292), anti-ICAM1 (R&D systems, AF796) and anti-cleaved caspase 3 (Cell Signaling Technology, #9661). Images were captured through a Nikon upright microscope with an objective set to ×4 or ×20 magnification, quantified using CellSens Standard software, and expressed as percent of plaque area (%) = (staining positive area/plaque area) × 100%.

### Serum lipid measurement

The mice were anesthetized with isoflurane (RWD, Life Science). Mouse blood was collected by heart puncture using 1 ml syringe treated with 0.9% saline supplemented with heparin (Wanbang, China) immediately prior to application. Serum was separated by centrifugation at 845 *g* for 5 min at RT. Serum lipid and lipoprotein profiles were measured per the manufacturer’s instructions (Leadman, China).

### Verhoeff elastic fiber staining

The processed paraffin sections were incubated in Verhoeff dye solution A (A1:A2:A3 = 5:2:2) for 20 min and rinsed with warm water. The sections were then stained with Verhoeff differentiation solution. Staining was monitored under microscope, and terminated once the elastic fiber turns black with a gray background. After washing with tap water, sections were quickly deiodized with 95% ethanol and counterstained with the VanGieson solution (C1:C2 = 1:9) for 3 min. After removing the excess staining solution, the sections were quickly dehydrated with absolute ethanol.

### Analysis of foam cell formation

BMDMs were seeded on 24-well plates (2.0 × 10^5^ cells/well) and incubated overnight. The cells were treated with or without 28 μg/mL of VX765 for 6 h and then loaded with oxLDL (Invitrogen) to a final concentration of 50 μg/mL for 24 h. Macrophages were fixed in 4% paraformaldehyde and stained for 30 min in 0.2% Oil Red O (in 60% isopropanol). The cells were examined under light microscopy, and ten representative images were captured for each condition in each experiment. The formation of foam cells was expressed as the percentage of Oil Red O-positive cells to total macrophages.

### Lactate dehydrogenase release assay

Lactate dehydrogenase (LDH) release cell death was determined by measuring LDH activity in the cell supernatants using a commercial cytotoxicity assay (Beyotime, Shanghai, China). In brief, macrophages seeded into 96 well plates (5 × 10^4^ cells/well) were treated as indicated in the Figure Legend and the supernatants collected. LDH released from the cells was measured using a Cytotoxicity Detection kit following the manufacturer’s protocol.

### Enzyme linked immunosorbent assay (ELISA)

The levels of IL-1β in cell culture medium and plasma were determined by ELISA kit (Neobioscience) according to the manufacturer’s instructions. For in vitro studies, culture media was collected immediately following the treatment of cells as indicated in the figure legend. Samples were cleared by centrifugation at 12,000 *g* for 5 min and the supernatants were stored at −20 °C prior to analysis.

### Determination of reactive oxygen species (ROS)

The content of intracellular ROS was detected by ROS assay kit (Beyotime, China) per the manufacturer’s instructions. Briefly, macrophages grown on the coverslips in 24-well plates were treated as indicated in the Figure Legend. The cells were then loaded with the dichlorofluorescin diacetate (DCFH-DA) (10 μM) in the presence of Mitotracker (100 nM) in serum-free medium in dark at 37 °C for 20 min, observed under a fluorescence microscope and images were captured.

### Hoechst 33342/propidium iodide (PI) staining

Macrophages cultured on the coverslips in 24-well plates were treated as specified in the Figure Legend. The cells were then loaded with a Hoechst 33342 (10 μg/mL) and PI (10 μg/mL) at 37 °C for 25 min to detect cell membrane integrity, visualized under a fluorescence microscope and images were captured.

### Detection of mitochondrial membrane potential (Δψm)

The Δψm was analyzed using the fluorescent probe JC-1, a dye that has two staining spectra, using JC-1 assay kit (Beyotime, China) according to the manufacturer’s instructions. Under normal conditions, JC-1 exhibits red fluorescence aggregates in the mitochondrial matrix. When the Δψm is reduced, monomeric JC-1 displays green fluorescence. In brief, macrophages cultured on the coverslips in 24-well plates were treated as detailed in the Figure Legend. The cells were loaded with JC-1 (1:400 dilution) at 37 °C for 20 min, observed under a fluorescence microscope and images were captured. A decline in red fluorescence intensity was considered an indicator of Δψm loss.

### Measurement of mitochondrial DNA (mtDNA) release

Macrophages were treated as detailed in the Figure Legend and harvested in 1% Igepal CA-630 (Sigma-Aldrich). After centrifugation at 16,000 *g* for 15 min at 4 °C, the cytosolic fraction was collected. The mtDNA in the cytosolic fraction was extracted with Cell & Tissue DNA Kit following the manufacturer’s protocol. Quantitative PCR was conducted to measure mtDNA encoding cytochrome c oxidase I and nuclear DNA encoding 18S ribosomal RNA. The following primers were used: cytochrome c oxidase I forward, 5′-GCCCCAGATATAGCATTCCC-3′, and reverse, 5′-GTTCATCCTGTTCCTGCTCC-3′; and 18S rDNA forward, 5′-TAGAGGGACAAGTGGCGTTC-3′, and reverse, 5′-CGCTGAGCCAGTCAGTGT-3′. The copy number of mtDNA was normalized to that of nuclear DNA.

### Subcellular fractionation

Cytoplasmic and mitochondrial proteins were extracted from macrophages using the Cytoplasmic and Mitochondrial Protein Extraction Kit (Sangon Biotech, Shanghai, China) following the manufacturer’s protocol. Briefly, macrophages were detached from the culture dishes and washed twice with ice-cold Phosphate-Buffered Saline (PBS, pH 7.4). Equal numbers of cells (1 × 10^7^/group) were resuspended into the cytoplasmic buffer with DTT, protease and phosphatase inhibitors. The cells were homogenized using Dounce homogenizer on ice. The homogenate was spun down at 800 *g* for 10 min at 4 °C. The supernatant was centrifuged at 12,830 *g* for 30 min at 4 °C. The resultant supernatant was saved as the cytoplasmic protein. The pellet was then resuspended into the mitochondria lysis buffer supplemented with DTT, protease and phosphatase inhibitors. After centrifuge at 15,000 *g* for 10 min at 4 °C, the supernatant was collected as the mitochondrial protein. The cytoplasmic and mitochondrial proteins were analyzed by immunoblotting.

### Animal treatment and characterization of atherosclerotic plaques

Animal studies were approved by the Animal Care and Use Committee from Renmin Hospital of the Hubei University of Medicine. *Ldlr*^*−/−*^, *ApoE*^*−/−*^, and *Nlrp3*^−/−^ mice on a C57BL/6 background were purchased from the Jackson Laboratories and maintained in specific pathogen free level, independent ventilation cage environment on a regular light-dark cycle (12 h light, 12 h dark). *Nlrp3*^−/−^ mice were crossed with *ApoE*^*−/−*^ mice to generate *Nlrp3*^−/−^*;ApoE*^*−/−*^ mice. Mice were randomly divided into two groups for each animal experiment. All mice were sex- and age-matched. Equal amounts of male and female animals were grouped randomly and used in each study. A total of 66 mice were used in our animal studies. To facilitate atherosclerotic lesion formation, 6–8-week-old male and female mice were placed on a high-fat diet (HFD) (D12079B, Research Diets). Mice were treated three times per week by intraperitoneal injection of 20% castor oil or VX765 (75 mg/kg in 20% castor oil) to a final volume of 100 μl for each mouse. At the end of the experiment, mice were anesthetized by isoflurane and blood collected from the left ventricle by cardiac puncture. The mice were perfused via the left ventricle with 0.9% saline supplemented with heparin (50 U/mL) and with 4% paraformaldehyde solution, respectively. The heart was harvested and embedded in paraffin or optimal cutting temperature compound (OCT, Tissue-Tek) and frozen in −80 °C for cryostats tissue sectioning. The entire aorta from the heart outlet to the iliac bifurcation was dissected, cleaned of adventitial and fat tissues, opened longitudinally, stained with Oil Red O, and pinned flat on a black wax surface as previously described [13]. Aorta images were captured through a stereomicroscope (Olympus SZX10) with a digital camera (Olympus). Plaque area was quantified using CellSens Standard software and expressed as percentage of stained area relative to total aortic area as suggested [21].

For aortic sinus analysis, the OCT-embedded aortas were sectioned sequentially beginning at the aortic valve. Sections were stained with Oil Red O. Mean lesion area (Oil Red O^+^ area) and necrotic area was calculated by measuring cross sections. Fibrous cap of the lesions was visualized with Verhöeff staining reagent (Leagene). Atherosclerotic lesion area and necrotic area were measured using CellSens Standard software. All quantifications were performed by two individuals in a blinded manner.

### Statistical analysis

Data analyses were performed using GraphPad Prism 8.0 (GraphPad Software). Normal distribution was evaluated using the Shapiro–Wilk test. The variance was similar between the groups. Two-tailed Student’s *t* test was applied to assess statistical differences between the two groups. Data are expressed as the mean ± SD. The values of *p* < 0.05 (*), *p* < 0.01 (**), *p* < 0.001 (***), and *p* < 0.0001 (****) were considered statistically significant.

## Results

### VX765 blunts the activation of the NLRP3 inflammasome

**Fig. 1:**
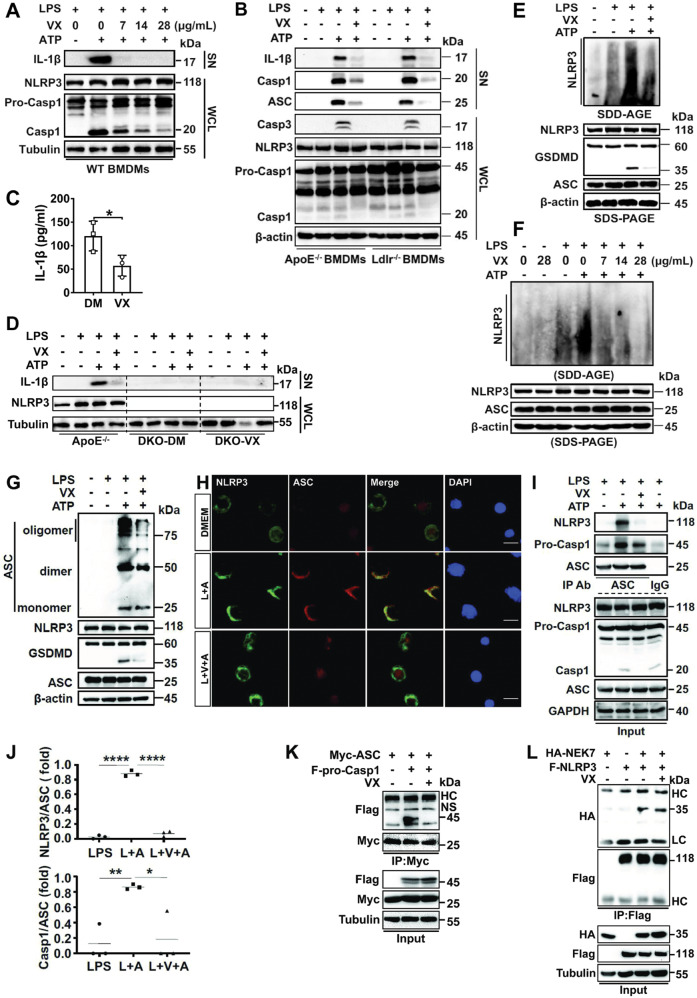
VX765 inhibits the activation and assembly of the NLRP3 inflammasome. **A**, **B** BMDMs obtained from C57BL/6 (WT), *ApoE*^−/−^ and *Ldlr*^−/−^ mice were treated with LPS (100 ng/mL, 8 h) and ATP (5 mM, 1 h) in the absence (−) or presence of VX765 (0, 7, 14 and 28 μg/mL; 2 h) (**A**) or VX765 (28 μg/mL, 2 h) (**B**). The supernatants and cell lysates were analyzed by immunoblotting. **C** J774A.1 macrophages were treated with LPS (100 ng/mL, 8 h), ATP (5 mM, 1 h) ± VX765 (28 μg/mL, 2 h). IL-1β in the supernatants was measured by ELISA (*n* = 3). **D** BMDMs isolated from *ApoE*^−/−^ or *Nlrp3*^−/−^*;ApoE*^−/−^ mice fed with high-fat diet ± VX765 (75 mg/kg) for 10 weeks were treated with without (−) or with LPS (100 ng/mL, 8 h), ATP (5 mM, 1 h) and VX765 (28 μg/mL, 2 h). Cell lysates were immunoblotted. **E**, **F** J774A.1 macrophages were treated with LPS (100 ng/mL, 8 h), ATP (5 mM, 1 h) ± 28 μg/mL of VX765 (**E**) or different amounts of VX765 (0, 7, 14 and 28 μg/mL) (**F**) for 2 h. Cell lysates were analyzed by SDD-AGE. **G** J774A.1 macrophages were treated as (**E**). ASC oligomerization was assessed. **H** J774A.1 macrophages were treated as (**E**). Double immunostaining was conducted with anti-NLRP3 and -ASC. The nuclei were stained with DAPI. **I**, **J** J774A.1 macrophages were treated as (**E**). Cell lysates were immunoprecipitated with anti-ASC or control IgG and immunoblotted with antibodies against NLRP3, procaspase 1 and ASC, respectively. Cell lysates were immunoblotted. For quantification analysis, gels were scanned and densitometry was performed using Image J (National Institutes of Health). The density of the NLRP3 or pro-caspase1 band was normalized to ASC, respectively, and expressed as a fold of the density measured (*n* = 3). **K** HEK293T cells transfected with the plasmids expressing myc-ASC and/or Flag-procaspase 1 were treated without or with VX765 (28 μg/mL, 16 h). Lysates were immunoprecipitated with anti-myc and immunoblotted with the indicated antibodies. **L** HEK293T cells were transfected with the vectors expressing HA-NEK7 and/or Flag-NLRP3 and treated without or with VX765 (28 μg/mL, 16 h). Lysates were immunoprecipitated with anti-Flag and then immunoblotted with the indicated antibodies. Cell lysates were analyzed by immunoblotting. LPS Lipopolysaccharides; SN supernatant; WCL whole cell lysates; BMDMs bone marrow-derived macrophages; WT wild-type; IL-1β interleukin 1β; pro-Casp 1 procaspase 1; Casp3 cleaved caspase 3; VX VX765; DM DMSO; HA hemagglutinin; F-NLRP3 Flag-NLRP3; F-Pro-Casp1 Flag-procaspase 1; DKO *Nlrp3*^−/−^*;ApoE*^−/−^ macrophages; HC heavy chain of immunoglobulin; LC light chain of immunoglobulin; NS non-specific band; IP immunoprecipitation; L + A LPS + ATP; L + V + A LPS + VX765 + ATP; kDa kilo-Dalton; SDD-AGE semi-denaturing detergent agarose gel electrophoresis; *p < 0.05; **p < 0.01; ****p < 0.0001.

We first determined the role for VX765 in regulating NLRP3 inflammasome activation in macrophages. As reported previously [13], treatment of wild-type (WT) macrophages with LPS + ATP induced NLRP3 inflammasome activation, as evidenced by caspase 1 activation and IL-1β release (Fig. 1A). Interestingly, VX765 curbed caspase 1 activation in a concentration-dependent manner (Fig. 1A). Notably, there was no discernible change in NLRP3 and pro-caspase 1 expression when different doses of VX765 were used, excluding the toxic effect of VX765. To verify and extend our observations, we conducted experiments with primary BMDMs from atherogenic mice. Also, to rule out the possibility that above findings can only be made in primary macrophages, we carried out some experiments with J774A.1 cells, a widely used macrophage cell line. Interestingly, similar findings were achieved with *ApoE*^−/−^ and *Ldlr*^−/−^ macrophages (Fig. 1B). ELISA confirmed the inhibitory role for VX765 in IL-1β release (Fig. 1C). ASC was reportedly found in the extracellular space, serving as a danger signal to propagate inflammation [13]. Indeed, VX765 dramatically abolished the release of ASC and active caspase 1 (Fig. 1B). However, LPS + ATP failed to induce IL-1β release in *Nlrp3*^−/−^*;ApoE*^−/−^ (Fig. 1D), *Nlrp3*^−/−^ (data not shown), and *Asc*-deficient RAW264.7 macrophages (Fig. 3E).

### VX765 antagonizes the assembly of the NLRP3 inflammasome

We tested the effect of VX765 on NLRP3 inflammasome assembly. VX765 robustly blunted NLRP3 oligomerization triggered by LPS + ATP in macrophages in a dose-dependent fashion (Fig. 1E, F). VX765 also curbed ASC oligomerization (Fig. 1G). Double immunostaining revealed an inhibitory effect of VX765 on co-localization of NLRP3 with ASC (Fig. 1H). Both NLRP3 and pro-caspase 1 were readily detected in the ASC immunoprecipitates (Fig. 1I, J). The NLRP3-ASC and ASC-pro-caspase 1 interactions were substantially compromised by VX765 (Fig. 1I, J). This finding was duplicated in the co-immunoprecipitation experiment with HEK293T cells (Fig. 1K). However, VX765 had no discernible effect on the NEK7-NLRP3 interaction (Fig. 1L). Together, VX765 antagonizes NLRP3 inflammasome assembly.

### VX765 blunts mitochondrial damage and promotes mitophagy

**Fig. 2:**
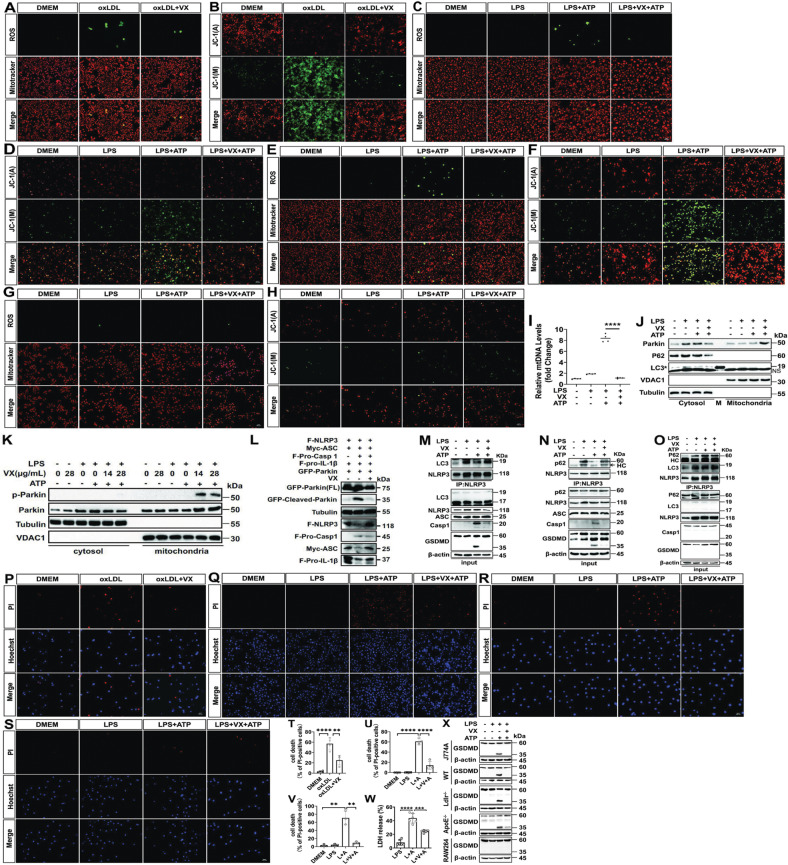
NLRP3 inflammasome activation amplifies mitochondrial damage and blocks mitophagy. **A**, **B** J774A cells were treated without or with oxLDL (100 μg/mL) ± VX765 (28 μg/mL) for 24 h. The cells were then stained with the dichlorofluorescin diacetate (DCFH-DA) and Mitotracker (**A**) and JC-1 (**B**). In the following experiments **C**–**K**, **M**–**O**, **Q**–**S**, macrophage were treated with DMEM (−), LPS (100 ng/mL, 8 h), and ATP (5 mM, 1 h) ± VX765 (28 μg/mL, 2 h) as indicated. **C**, **E**, **G** WT (**C**), J774A.1 (**E**) or RAW264.7 (**G**) macrophages were treated and stained with DCFH-DA and Mitotracker. **D**, **F**, **H** WT (**D**), J774A.1 (**F**) or RAW264.7 (**H**) macrophages were treated and stained with JC-1. **I** mitochondrial cytochrome c oxidase I DNA release in immortalized BMDMs were analyzed by qPCR, normalized to nuclear DNA encoding 18S ribosomal RNA (internal control), and expressed as fold change. **J**, **K** J774A.1 cells were treated as indicated. The mitochondrial and cytosolic proteins were prepared and examined by immunoblotting. **L** HEK293T cells were transfected with the plasmids expressing Flag-NLRP3, myc-ASC, Flag-Pro-IL-1β, GFP-Parkin, together with (+) or without (−) of vector expressing Flag-Pro-Casp1. The cell lysates were analyzed by immunoblotting. **M–O** J774A.1 (**M**, **N**) and RAW264.7 cells (**O**) were treated as indicated. Immunoprecipitation was conducted with anti-NLRP3 and then immunoblotted with anti-LC3 or p62. Cell lysates were probed by immunoblotting. **P** PI and Hoechst 33342 co-staining was conducted in J774A.1 macrophages treated without or with oxLDL (100 μg/mL) ± VX765 (28 μg/mL) for 24 h. **Q**–**S** PI and Hoechst 33342 co-staining was conducted in WT (**Q**), J774A.1 (**R**) and RAW264.7 **S** macrophages treated as indicated. **T**–**V** Quantification of PI positive cells shown in panels **Q**–**S**. **W** J774A.1 macrophages were treated with LPS (100 ng/mL, 8 h), and ATP (5 mM, 1 h) ± VX765 (28 μg/mL, 2 h). The supernatants were collected to measure the content of LDH (*n* ≥ 3). **X** Different types of macrophages were treated as (**W**) and gasdermin D processing was analyzed by immunoblotting. oxLDL oxidized LDL; ROS reactive oxygen species; LDH lactate dehydrogenase; DM DMSO; VX VX765; LPS Lipopolysaccharides; PI Propidium iodide; L + A LPS + ATP; L + V + A LPS + VX765 + ATP; GSDMD gasdermin D; p-Parkin phosphorylated Parkin; WT wild-type; mtDNA mitochondrial DNA; LC3 microtubule-associated protein light chain 3; VDAC1 voltage-dependent anion channel 1; Pro-IL-1β pro-interleukin 1β; Pro-Casp1 pro-caspase1; Casp1 cleaved caspase 1; VX VX765; HC heavy chain of immunoglobulin; FL full-length; NS non-specific band; M protein marker; kDa kilo-Dalton; **p < 0.01; ***p < 0.001; ****p < 0.0001.

We explored how VX765 counteracts NLRP3 inflammasome assembly. Whether NLRP3 inflammasome activation induces mitochondrial damage is unclear. Thus, we investigated whether caspase 1 inhibition mitigates mitochondrial damage and promotes mitophagy. Exposure of macrophages to oxLDL induced mitochondrial damage, as indicated by an increase in mitochondrial ROS (mROS) production and dissipation of mitochondrial membrane potential, both of which were antagonized by VX765 (Fig. 2A, B). VX765 alleviated mitochondrial damage induced by treatment of WT (Fig. 2C, D) and J774A.1 (Fig. 2E, F) macrophages with LPS + ATP. Nonetheless, mitochondrial damage was not observed in *Asc*-deficient RAW264.7 cells (Fig. 2G, H). VX765 alone did not give rise to mitochondrial damage (Supplementary Fig. 1A, B). Mitochondrial damage induces cytosolic release of mitochondrial DNA (mtDNA) [22]. mtDNA associates with NLRP3, promoting NLRP3 inflammasome assembly and activation [6, 23, 24]. qPCR showed that VX765 robustly repressed cytosolic release of mtDNA in macrophages induced by LPS + ATP (Fig. 2I). Together, NLRP3 inflammasome activation amplifies mitochondrial damage and such damage can be potently abrogated by caspase 1 inhibition.

Mitophagy functions to eliminate damaged mitochondria, which can be compromised by activated caspase 1 [25]. Parkin, a central regulator of mitophagy, is recruited to damaged mitochondria to initiate mitophagy [25]. Subcellular fractionation demonstrated that VX765 treatment increased the levels of mitochondrial Parkin but reduced p62 in the cytosol (Fig. 2J). However, there was minimal, if any, change in LC3 protein abundance (Fig. 2J). This is largely due to the fact that LC3I is cytosolic and LC3II is associated with autophagosomal membrane [26]. Upon mitochondrial damage, PTEN-induced putative kinase 1 (PINK1), a mitochondrial kinase, facilitates mitochondrial recruitment and phosphorylation at Serine 65 of Parkin [27]. Importantly, VX765 pronouncedly enhanced Parkin phosphorylation (Fig. 2K). Loss of Parkin enhances mitochondrial damage [22]. VX765 did not significantly alter Parkin expression (data not shown). Parkin is a substrate for caspase 1 [28]. We asked whether VX765 hinders Parkin cleavage by active caspase 1 using the HEK293T reconstitution system. Caspase 1 potently cleaved Parkin upon NLRP3 inflammasome activation, which was counteracted by VX765 (Fig. 2L). Together, VX765 dampens Parkin cleavage and promotes its mitochondrial translocation and phosphorylation.

Given that autophagy can physically interact with and subsequently degrade inflammasome components [29], we examined the impact of VX765 on the interaction of autophagy components (p62 and LC3) with NLRP3. VX765 promoted the interaction of NLRP3 with both LC3 and p62 in J774A.1 (Fig. 2M, N) but not RAW264.7 cells (Fig. 2O). Together, VX765 reverts mitophagy by blunting activated caspase 1-induced mitochondrial damage.

### VX765 restricts pyroptotic cell death of macrophages

Mitochondrial damage precipitates pyroptosis [30]. Pyroptosis is characterized by loss of plasma membrane integrity and release of intracellular contents [31]. OxLDL enhanced propidium iodide (PI)/Hoechst 33342 uptake in J774A.1 macrophages, which was significantly blocked by VX765 (Fig. 2P, T). Likewise, VX765 robustly suppressed PI influx in WT (Fig. 2Q, U) and J774A.1 (Fig. 2R, V) macrophages triggered by LPS + ATP. Importantly, VX765 potently abrogated lactate dehydrogenase (LDH) release in J774A.1 macrophages ignited by LPS + ATP (Fig. 2W). The similar findings were made in immortalized BMDMs (Supplementary Fig. 2A, C). However, VX765 alone had no significant influence on PI intake (Supplementary Fig. 1C). Exposure of RAW264.7 macrophages to LPS + ATP did not influence PI influx (Fig. 2S).

Activated caspase 1 cleaves GSDMD to produce active N-terminal fragment of GSDMD, forming pores in the plasma membrane to trigger pyroptosis [32]. As expected, LPS + ATP pronouncedly triggered GSDMD cleavage in WT, J774A.1, *Ldlr*^−/−^ and *ApoE*^−/−^ macrophages, which was abolished by VX765 (Fig. 2X). In contrast, GSDMD cleavage was not seen in *Asc*-deficient RAW264.7 (Fig. 2X) and *Nlrp3*^−/−^ (data not shown) macrophages. Together, caspase 1 inhibition mitigated NLRP3 inflammasome activation-licensed pyroptosis.

### VX765 attenuates caspase 3 activation

**Fig. 3:**
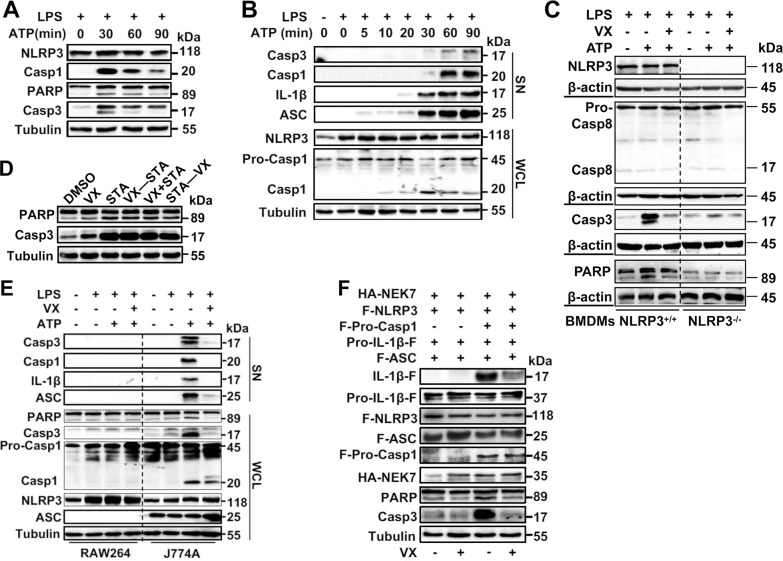
VX765 impedes macrophage pyroptosis. **A**, **B** J774A.1 macrophages were treated with LPS (100 ng/mL, 8 h) and ATP (5 mM) for different time points (0–90 min). Cell lysates and supernatants were analyzed by immunoblotting. **C** *Nlrp3*^−/−^ and *Nlrp3*^*+/+*^ BMDMs were treated with LPS (100 ng/mL, 8 h), ATP (5 mM) ± VX765 (28 μg/mL, 2 h). Cell lysates were examined by immunoblotting. **D** J774A.1 macrophages were treated with staurosporine (1 μM) for 4 h. VX765 (28 μg/mL, 2 h) was added before, after or simultaneously with staurosporine as indicated. **E** RAW264.7 and J774A.1 macrophages were treated as (**C**). Supernatants and cell lysates were monitored by immunoblotting. **F** HEK293T cells were transfected with the plasmids expressing HA-NEK7, Flag-NLRP3, Pro-IL-1β-Flag, Flag-ASC, together with (+) or without (−) of vector expressing Flag-procaspase 1. The cells were treated without or with VX765 (28 μg/mL, 16 h). Cell lysates were analyzed by immunoblotting. PARP poly(ADP-ribose) polymerase; IL-1β interleukin 1β; Casp8 cleaved caspase 8; Pro-Casp8 procaspase 8; Casp3 cleaved caspase 3; Casp1 cleaved caspase 1; Pro-Casp1 procaspase 1; VX VX765; STA staurosporine; F-NLRP3 Flag-NLRP3; F-Pro-Casp1 Flag-procaspase 1; Pro-IL-1β-F pro-interleukin 1β-Flag; F-ASC Flag-ASC; HA hemagglutinin; LDH Lactate dehydrogenase; SN supernatants; WCL whole cell lysates; M_Φ_ macrophages; kDa kilo-Dalton; ***p < 0.001; ****p < 0.0001.

An apoptotic program is readily activated upon NLRP3 inflammasome activation with ill defined mechanism [33]. As expected, treatment of macrophages with LPS + ATP activated caspase 3, as judged by the cleavage of procaspase 3 and its substrate PARP in J774A.1 (Fig. 3A, E), WT (Fig. 3C), *ApoE*^−/−^ (Fig. 1B) and *Ldlr*^−/−^ (Fig. 1B) macrophages. In contrast, caspase 3 activation was not seen in *Nlrp3*^−/−^ (Fig. 3C) and *Asc*-null RAW264.7 (Fig. 3E) macrophages. Cleaved caspase 3 was also found in the supernatants (Fig. 3B, E). However, VX765 had no significant impact on caspase 3 activation induced by staurosporine (Fig. 3D), a well-known caspase 3 activator [34]. Also, there was no discernible difference in caspase 8 activation in *Nlrp3*^+/+^ and *Nlrp3*^−/−^ macrophages treated with LPS + ATP compared with those exposed to LPS alone (Fig. 3C). To further ascertain whether VX765 impeded caspase 3 activation through targeting active caspase 1, we conducted reconstitution of the NLRP3 inflammasome in HEK293T cells, a widely used approach to probe inflammasome regulation [13]. Reconstitution of the NLRP3 inflammasome in HEK293T induced caspase 1 activation and IL-1β production (Fig. 3F). Endogenous caspase 3 was robustly activated in reconstituted cells in the presence but not absence of procaspase 1 (Fig. 3F). Moreover, VX765 blocked PARP cleavage (Fig. 3F). Collectively, caspase 3 is activated in macrophages as a result of NLRP3 inflammasome activation.

### VX765 suppresses macrophage foam cell formation through repressing NLRP3 inflammasome activation

**Fig. 4:**
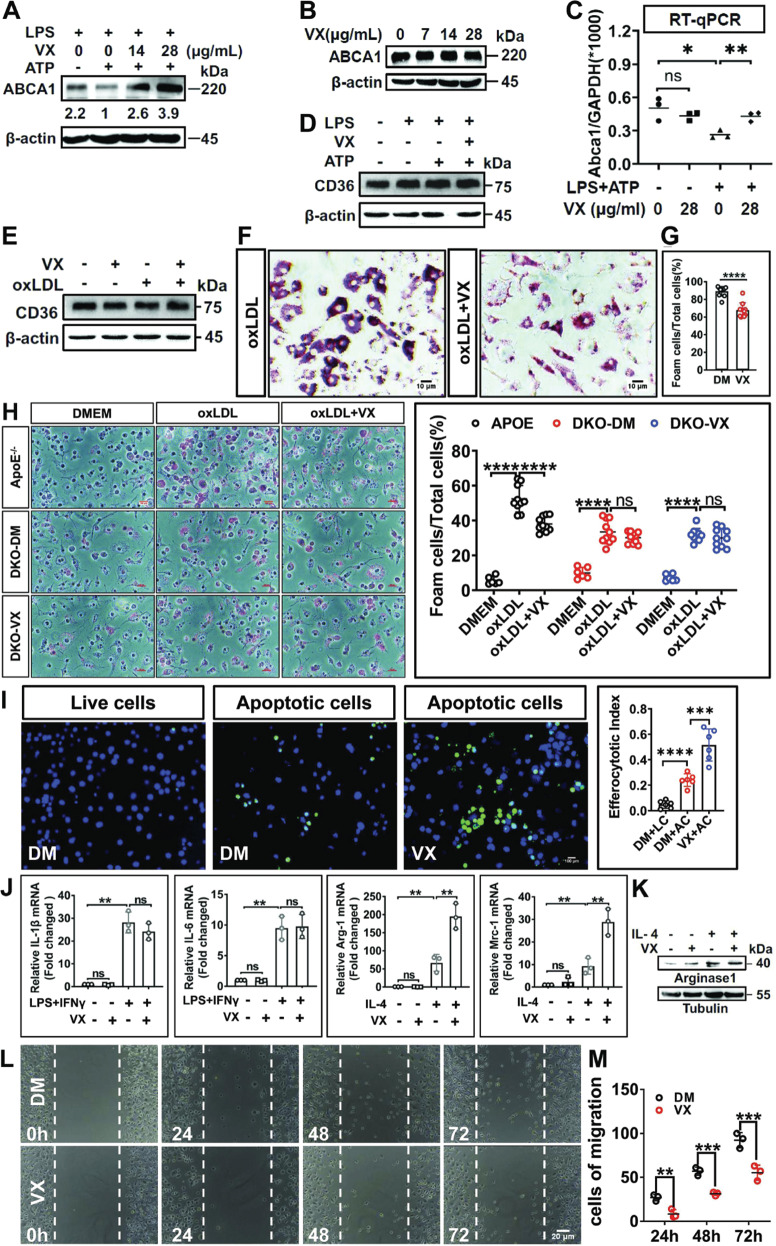
The impact of VX765 on the properties of macrophages associated with atherosclerosis. **A** Immunoblotting analysis of ABCA1 expression in J774A.1 macrophages treated with DMEM (−), LPS (100 ng/mL, 8 h), ATP (5 mM, 1 h), and VX765 (0, 14 and 28 μg/mL; 2 h). **B** Immunoblotting analysis of ABCA1 expression in J774A.1 macrophages treated with different doses of VX765 (0, 7, 14 and 28 μg/mL; 2 h). **C** RT-qPCR analysis of *Abca1* mRNA in J774A.1 macrophages treated without or with LPS (100 ng/mL, 8 h) + ATP(5 mM, 1 h) ± VX765 (28 μg/mL, 2 h). **D** Immunoblotting analysis of CD36 expression in BMDMs treated as (**C**). **E** Immunoblotting analysis of CD36 expression in BMDMs treated with oxLDL (100 μg/mL), VX765 (28 μg/mL), alone or together, for 24 h. **F–H** BMDMs from WT mice (**F**, **G**), *ApoE*^−/−^ or DKO (*Nlrp3*^−/−^*;ApoE*^−/−^) mice fed with high-fat-diet ± VX765 (75 mg/kg) for 12 weeks (**H**) were loaded with oxLDL (100 μg/mL) ± VX765 (28 μg/mL) for 24 h. Foam cells formation was visualized by Oil red O staining. The percentage of Oil red O-positive cells to total cells of ten high-power fields were calculated (*n* ≥ 3). **I** The live or apoptotic Jurkat cells labeled with Calcein were loaded to WT BMDMs ± VX765 (28 μg/mL, 2 h). The efferocytotic cells were visualized and quantified. The percentage of efferocytotic cells to total macrophages of 10 high-power fields were calculated. **J** J774A.1 macrophages were treated with LPS (100 ng/mL) and INFγ (50 ng/mL) ± VX765 (28 μg/mL) or IL-4 (20 ng/mL) ± VX765 (28 μg/mL) for 24 h. RT-qPCR was conducted to measure the expression of the indicated genes. Shown is a fold change of the expression of the indicated gene to *GAPDH* mRNA. **K** J774A.1 macrophages were treated without or with IL-4 (20 ng/mL) and/or VX765 (28 μg/mL) for 24 h. Immunoblotting was performed to examine the expression of the indicated proteins. **L**, **M** Wound assay was carried out to determine the role for VX765 (28 μg/mL, 0–72 h) in macrophage migration. J774A.1 macrophages were scratched and the cells in the denuded regions were counted. BMDMs bone marrow-derived macrophages; oxLDL oxidized low-density lipoproteins; DKO *Nlrp3*^−/−^*;ApoE*^−/−^ macrophages; DM DMSO; VX VX765; IL-4 interleukin 4; AC apoptotic cells; LC live cells; kDa kilo-Dalton; ns no significance; **p < 0.01; ***p < 0.001; ****p < 0.0001.

Deregulation of cholesterol efflux precipitates the formation of foam cells [35]. Cholesterol efflux is meditated largely by ATP-binding cassette transporters (ABCs, e.g., ABCA1) while macrophages take up oxLDL primarily through scavenger receptor CD36 [35]. We tested the effect of VX765 on CD36 and ABCA1 expression and foam cell formation. Treatment with LPS + ATP declined ABCA1 expression in J774 A.1 (Fig. 4A), *ApoE*^−/−^ (Supplementary Fig. 3) and WT (data not shown) macrophages. Importantly, VX765 markedly reverted ABCA1 reduction in a dose-dependent manner (Fig. 4A). Given that IL-1β declined the levels of *Abca1* mRNA in macrophages [36], we tested whether VX765 affected the expression of *Abca1* mRNA. Exposure of macrophages to LPS + ATP significantly suppressed *Abca1* mRNA levels, which was greatly antagonized by VX765 (Fig. 4C). Notably, VX765 alone had no discernible effect on ABCA1 expression (Fig. 4B, C). In contrast, VX765 had no significant impact on CD36 expression (Fig. 4D, E). As expected, VX765 repressed oxLDL-laden macrophage foam cell formation (Fig. 4F, G). Importantly, VX765 suppressed foam cell formation in *ApoE*^−/−^ but not in *Nlrp3*^−/−^*;ApoE*^−/−^ macrophages (Fig. 4H). Our study supports that NLRP3 inflammasome activation facilitates foam cell formation [36]. Therefore, VX765 inhibits foam cell formation, at least partially, through repressing NLRP3 inflammasome activation.

### VX765 regulates efferocytosis, M2 polarization and migration of macrophages

We evaluated the effect of VX765 on efferocytosis. VX765 significantly enhanced the ability of macrophages to ingest apoptotic T lymphocytes (Fig. 4I). Efferocytosis reprograms macrophages toward an M2 phenotype [37]. Macrophages are broadly classified into M1 and M2 subtypes [38]. M2 macrophages promote inflammation resolution [38]. Importantly, VX765 potentiated IL4-induced M2 polarization, as indicated by increased expression of Mrc-1 and arginase 1 (Fig. 4J, K), two well-recognized markers of M2 macrophages [39]. In contrast, VX765 did not alter M1 polarization (Fig. 4J).

Both macrophage migration [40] and proliferation [41] contribute to macrophage expansion in atherosclerotic plaques. We examined the effect of VX765 on migration and proliferation of macrophages. Wound-healing assay indicated that VX765 impaired the ability of macrophages to migrate into the denuded region (Fig. 4L, M). However, VX765 was ineffective in macrophage proliferation (data not shown). Collectively, VX765 influences multiple properties of macrophages involved in vascular inflammation and atherosclerosis.

### VX765 ameliorates vascular inflammation and atherosclerosis in *Ldlr*-deficient mice

**Fig. 5:**
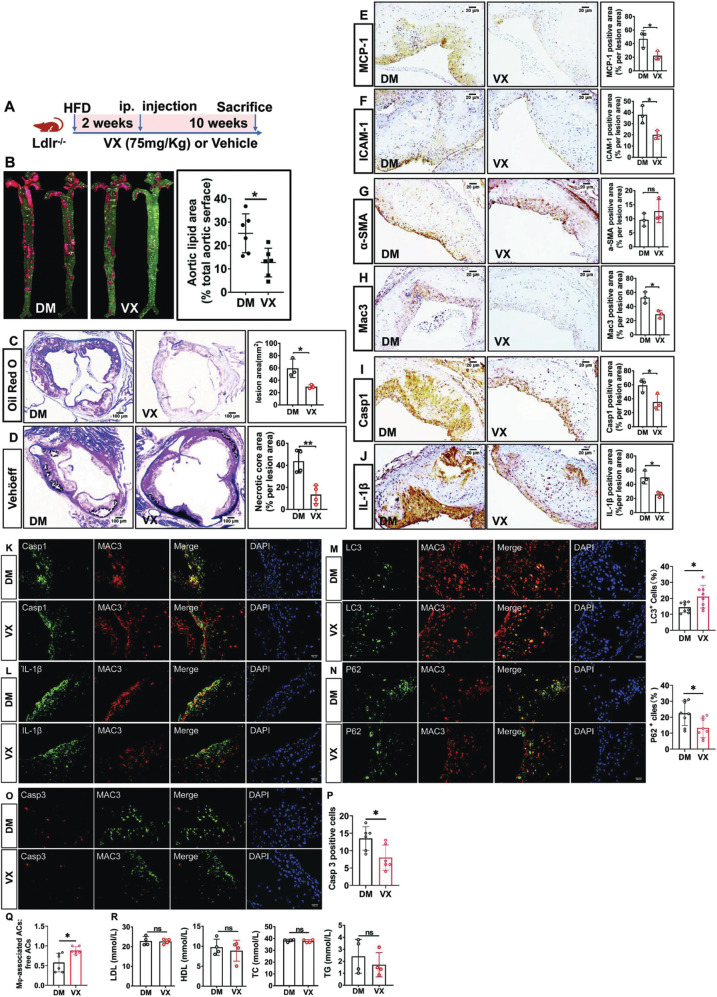
VX765 restrains vascular inflammation and atherosclerosis in low-density lipoprotein receptor–deficient (*Ldlr*^*−/−*^) mice. **A** Diagramed is the regimen for treatment of *Ldlr*^−/−^ mice (6–8 weeks old) with VX765 (VX, *n* = 6) or vehicle (DM, *n* = 6). **B**, **C** The atherosclerotic lesions in the entire aortas (*n* = 6) and the aortic sinuses were visualized by oil red O staining. **D** The necrotic areas were visualized by Verhöeff staining. **E**–**J** The expression of the indicated proteins in the aortic sinuses was detected by immunohistochemical staining. The positive area of each protein was measured. **K**–**N** Dual immunofluorescence staining of the indicated proteins in the aortic sinuses. **O**–**Q** The expression of Caspase 3 and Mac3 was stained (**O**) and quantitated to assess cell death (**P**) and in situ macrophage efferocytosis (**Q**). **R** Profile of lipids and lipoproteins (*n* ≥ 3). HFD high-fat diet; ip intraperitoneal; LDL low-density lipoprotein; HDL high density lipoprotein; TC total cholesterol; TG total triglyceride; DM DMSO; VX VX765; LC3 microtubule-associated protein light chain 3; α-SMA α-smooth muscle actin; MCP-1 monocyte chemoattractant protein-1; ICAM-1 intercellular cell adhesion molecule 1; Casp1 cleaved caspase 1; Casp3 cleaved caspase 3; IL-1β interleukin 1β; ACs apoptotic cells; ns no significance; *p < 0.05; **p < 0.01.

To probe the biological relevance of our mechanistic findings, we dissected the effect of VX765 on vascular inflammation and atherosclerosis. We first gauged the significance of VX765 administration in *Ldlr* ^*−/−*^ mice. *Ldlr* ^*−/−*^ mice were placed on a HFD for 2 weeks, and then administrated with VX765 while continuing to feed a HFD for an additional 10 weeks (Fig. 5A). The atherosclerotic lesions in the entire aortas (Fig. 5B) and the aortic roots (Fig. 5C) from VX765-treated mice were markedly declined compared to those in control group. Treatment with VX765 reduced the necrotic core in the plaques (Fig. 5D).

The development of atherosclerosis begins with monocyte recruitment into the arterial walls [3]. Monocyte chemoattractant protein-1 (MCP-1) and intercellular cell adhesion molecule 1 (ICAM-1) are critical for monocyte recruitment [3]. The expression levels of ICAM-1 and MCP-1 were significantly declined in the plaques of VX765-treated mice compared with control mice (Fig. 5E, F). The content of macrophages but not smooth muscle cells (SMCs) in the plaques was markedly reduced by VX765 treatment (Fig. 5G, H). Notably, staining with normal IgG demonstrated the specificity of immunohistochemical results (data not shown).

We then assessed the role of VX765 in manipulating NLRP3 inflammasome activation in the plaques by immunohistochemical staining with anti-caspase 1 that is selectively recognized active caspase 1. Compared to control mice, VX765-treated mice exhibited a significant inhibition of NLRP3 inflammasome activation in the lesions, as indicated by reduced IL-1β expression and caspase 1 activation (Fig. 5I, J). Furthermore, NLRP3 inflammasome activation in lesional macrophages (Fig. 5K, L) but not SMCs (Supplementary Fig. 4) was markedly attenuated by VX765.

Autophagy and the NLRP3 inflammasome are mutually exclusive [6, 7, 29, 42]. Autophagy protects against vascular inflammation and atherosclerosis [43]. VX765 treatment enhanced LC3 expression in the lesions (Fig. 5M) but decreased p62 levels (Fig. 5N), supporting that VX765 treatment promoted autophagy in the lesions. Effective efferocytosis inhibits inflammation and retards necrotic core formation [44]. VX765 administration repressed lesional cell death, as demonstrated by a decrease in cleaved caspase 3 expression (Fig. 5O, P). Treatment with VX765 substantially facilitated macrophage efferocytosis (Fig. 5O, Q). No overt change in the levels of lipids and lipoproteins between two groups was seen (Fig. 5R). Together, VX765 restrains vascular inflammation and atherosclerosis in *Ldlr* ^*−/−*^ mice.

### VX765 mitigates vascular inflammation and atherosclerosis in *ApoE*-deficient mice

**Fig. 6:**
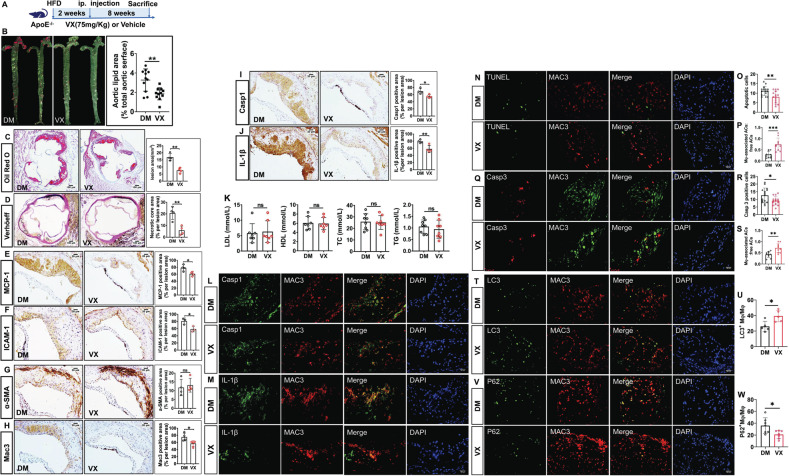
VX765 restricts vascular inflammation and atherosclerosis in apolipoprotein E–deficient (*ApoE*^*−/−*^) mice. **A** Diagramed is the regimen for treatment of *ApoE*^*−/−*^ mice (6–8 weeks old) with VX765 (VX, *n* = 11) or vehicle (DM, *n* = 11); **B**, **C** The atherosclerotic lesions in the whole aortas (*n* = 11) and the aortic roots (*n* = 4) were visualized by oil red O staining. **D** The necrotic regions were visualized by Verhöeff staining. **E**–**J** The expression of the indicated proteins in the aortic sinuses was detected by immunohistochemical staining. The positive area of each protein was measured. **K** The profile of lipids and lipoproteins (*n* = 3). **L**, **M** Double immunostaining of the indicated proteins in the aortic sinuses. **N**–**S** The expression of Caspase 3 and Mac3 was stained and quantitated to assess cell death and in situ macrophage efferocytosis. **T**–**W** Macrophage autophagy was assessed by immunostaining with the indicated antibodies and quantitated. HFD high-fat diet; ip intraperitoneal; LDL low-density lipoprotein; HDL high density lipoprotein; TC total cholesterol; TG total triglyceride; α-SMA α-smooth muscle actin; MCP-1 monocyte chemoattractant protein-1; ICAM-1 intercellular cell adhesion molecule 1; IL-1β interleukin 1β; Casp1 cleaved caspase 1; Casp3 cleaved caspase 3; ACs apoptotic cells; DM DMSO; VX VX765; M_Φ_ macrophages; LC3 microtubule-associated protein light chain 3; ns no significance; *p < 0.05; **p < 0.01; ***p < 0.001.

**Fig. 7:**
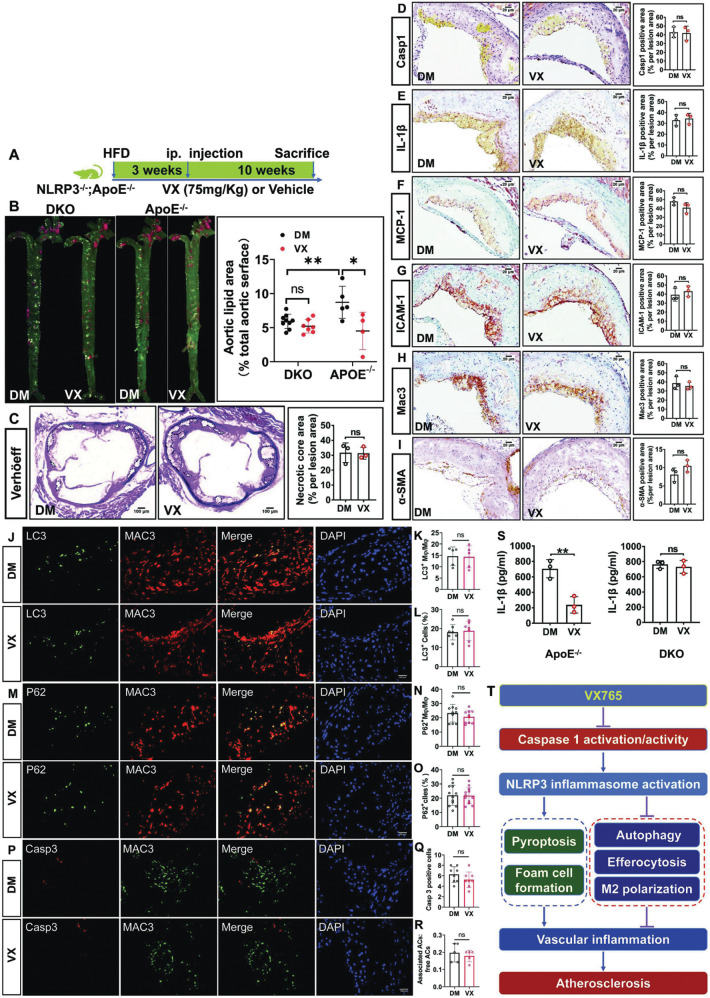
NLRP3 deficiency impairs the ability of VX765 to manipulate diet-induced vascular inflammation and atherosclerosis in *ApoE*^−/−^ mice. **A** Diagramed is the regimen for treatment of *ApoE*^−/−^ mice with VX765 (VX, *n* = 4) or vehicle (DM, *n* = 5) and *Nlrp3*^−/−^*;ApoE*^−/−^ mice with VX765 (VX, *n* = 7) or vehicle (DM, *n* = 9). **B** The atherosclerotic lesions in the whole aortas were visualized by oil red O staining (*n* ≥ 3). **C** The necrotic regions in the aortic sinuses were visualized by Verhöeff staining (*n* ≥ 3). **D**–**I** The expression of the indicated proteins in the aortic sinuses was determined by immunohistochemical staining. The positive area of each protein was measured (*n* ≥ 3). **J**–**O** Immunostaining analysis of the aortic sinuses with the indicated antibodies. Lesional macrophage expression of LC3 and p62 were quantitated. **P**–**R** The expression of caspase 3 and Mac3 was stained and quantitated to measure cell death and in situ macrophage efferocytosis. **S** Measurement of IL-1β content in the blood from *ApoE*^−/−^ and *Nlrp3*^−/−^*ApoE*^−/−^ mice treated as indicated in (**A**) by ELISA (*n* = 3). **T** Schematic model for the role and mechanisms of VX765 action. DKO *Nlrp3*^−/−^*;ApoE*^−/−^ mice; HFD high-fat diet; ip intraperitoneal; DM DMSO; VX VX765; α-SMA α-smooth muscle actin; MCP-1 monocyte chemoattractant protein-1; ICAM-1 intercellular cell adhesion molecule 1; LC3 microtubule-associated protein light chain 3; IL-1β interleukin 1β; M_Φ_ macrophage; ACs apoptotic cells; ns no significance; *p < 0.05; **p < 0.01.

To determine the role of VX765 in vascular inflammation and atherosclerosis in *ApoE*^*−/−*^ mice, mice were treated as depicted in Fig. 6A. The VX765-treated mice displayed a marked reduction in the lesions in the whole aortas (Fig. 6B) and the aortic roots (Fig. 6C) compared to vehicle-treated *ApoE*^*−/−*^ mice. The necrotic core in the plaque in VX765-treated mice was much smaller than that in control mice (Fig. 6D). VX765 treatment led to a significant reduction in both MCP-1^+^ area (Fig. 6E) and ICAM-1^+^ area (Fig. 6F) in the lesions. VX765-treated mice exhibited a marked decrease in lesional infiltration of macrophages in comparison to vehicle-treated *ApoE*^*−/−*^ mice (Fig. 6H). There was no significant difference in the content of SMCs between two groups (Fig. 6G). VX765 treatment markedly suppressed caspase 1 activation (Fig. 6I) and IL-1β expression (Fig. 6J) in the lesion in VX765-treated mice compare with control mice. Strikingly, IL-1β content in the blood from VX765-treated mice was significantly lower than vehicle-treated mice (Fig. 7S). VX765 had no significant impact on the levels of plasma lipids and lipoproteins (Fig. 6K). Both caspase 1 activation (Fig. 6L) and IL-1β expression (Fig. 6M) were found predominantly in macrophages rather than SMCs (data not shown). As expected, VX765 treatment markedly suppressed macrophage caspase 1 activation (Fig. 6L) and IL-1β expression (Fig. 6M).

Strikingly, VX765 treatment suppressed cell death in the plaques, as demonstrated by decrease in TUNEL^+^ staining (Fig. 6N, O) and cleaved caspase 3 expression (Fig. 6Q, R) in the aortic sinuses. VX765 administration enhanced macrophage efferocytosis (Fig. 6N, P, Q, and S). Importantly, VX765 enhanced LC3 expression in macrophages (Fig. 6T, U) but suppressed p62 expression (Fig. 6V, W) in the aortic sinuses. Thus, treatment with VX765 markedly promoted macrophage autophagy. Collectively, VX765 efficiently alleviates vascular inflammation and atherosclerosis.

### Ablation of NLRP3 counteracts the ability of VX765 to restrict vascular inflammation and atherosclerosis in *ApoE*-deficient mice

We substantiated if VX765 exerts its in vivo functions, at least partially, through targeting NLRP3 inflammasome activation. We generated *Nlrp3*^−/−^*;AopE*^−/−^ double knockout mice for evaluating the role of VX765 in this model (Fig. 7A). NLRP3 was detectable in *AopE*^−/−^ but not in *Nlrp3*^−/−^*;AopE*^−/−^ mice (Fig. 1D and Supplementary Fig. 5). The atherosclerotic lesions in VX765-treated *AopE*^−/−^ mice were significantly reduced (Fig. 7B).VX765 had no significant effect on the formation of atherosclerotic lesions in *Nlrp3*^−/−^*;AopE*^−/−^ mice compared with those in vehicle-treated group (Fig. 7B). Compared to the vehicle treatment, VX765 administration had no significant impact on the necrotic size (Fig. 7C), on the expression of active caspase 1 (Fig. 7D), IL-1β (Fig. 7E), MCP-1 (Fig. 7F) and ICAM-1(Fig. 7G), as well as on the content of lesional macrophages (Fig. 7H) and SMCs (Fig. 7I). VX765 treatment of *Nlrp3*^−/−^*;AopE*^−/−^ mice failed to alter lesional expression of LC3 (Fig. 7J–L) and p62 (Fig. 7M–O). Likewise, administration of *Nlrp3*^−/−^*;AopE*^−/−^ mice with VX765 had no significant impact on macrophage death (Fig. 7P, Q) and efferocytosis (Fig. 7P, R) when compared to vehicle treatment. VX765 markedly reduced blood levels of IL-1β in *ApoE*^−/−^ mice but not *Nlrp3*^−/−^*;AopE*^−/−^ mice (Fig. 7S). Collectively, VX765 functions in mitophagy, efferocytosis, vascular inflammation and atherosclerosis were greatly compromised in *Nlrp3*^−/−^*;AopE*^−/−^ mice.

## Discussion

This study aimed to delve into the mechanism behind the NLRP3 inflammasome in dictating atherosclerosis and to explore the role and mechanism for VX765 in targeting atherosclerosis. Our animal study clarified that NLRP3 inflammasome activation promotes vascular inflammation and atherosclerosis in both *Ldlr*^*−/−*^ and *ApoE*^*−/−*^ mice. During the course of our study, a report described that VX765 impeded atherosclerosis in *ApoE*^−/−^ mice [45]. In addition, a genetic study documented that *caspase 1* deficiency ameliorated atherosclerosis in *ApoE*^−/−^ mice [46]. It was unknown whether VX765 was athero-protective in *Ldlr*^*−/−*^ mice. Also, the mechanism that caspase 1 inhibition/deletion mitigates atherosclerosis is unclear. Our work revealed that VX765 promoted autophagy and efferocytosis, hindered cell death, and restrained vascular inflammation and atherosclerosis in both *Ldlr*^*−/−*^ and *ApoE*^*−/−*^ mice. Ablation of NLRP3 compromised VX765 role in suppressing vascular inflammation and atherosclerosis. Future study is merited to substantiate whether more robust caspase 1-inhibitory effect could be achieved when VX765 is administrated at a higher dose or for a longer duration. These animal findings provided strong support for our in vitro studies showing that VX765 regulates NLRP3 inflammasome activation-associated events (Fig. 7T). Our data highlight that VX765 functions largely through targeting the NLRP3 inflammasome. Undoubtedly, our work significantly extends previous findings about caspase 1 in atherosclerosis [45, 46]. Moderate caspase 1 activation occurred in *Nlrp3*-deficient *ApoE*^−/−^ mice. It is likely that the atherogenic factors may promote the activation of other inflammasomes in atherosclerotic lesions. In support of our surmise, the NLRP1 inflammasome was significantly up-regulated in human atherosclerotic lesions [47]. Moreover, a recent study on the implication of AIM2 inflammasome to atherosclerosis reveals new mechanism for clonal hematopoiesis-associated atherosclerosis [48]. Therefore, the role of other types of inflammasomes in atherosclerosis warrants to be investigated.

Beyond the role for VX765 in curbing caspase 1 activity [11], this study was the first to assign VX765 an unexpected function in counteracting NLRP3 inflammasome assembly, a prerequisite for inflammasome activation [3]. Our work highlights that VX765 not only inhibits caspase 1 activity but also suppresses its activation. Importantly, VX765 has potential to protect against mitochondrial damage, as assessed by reduced mROS production and cytosolic release of mtDNA. ROS induces mtDNA oxidization; oxidized mtDNA interacts with NLRP3, contributing to NLRP3 inflammasome assembly and activation [6, 23, 24]. Our work showed that active caspase 1 promoted Parkin cleavage and that NLRP3 inflammasome activation impairs mitophagy. We showed that VX765 restricts cleavage of Parkin by caspase 1 and potentiates mitophagy, thus impeding NLRP3 inflammasome assembly and activation. Collectively, VX765 antagonizes NLRP3 inflammasome assembly, at least partially, by alleviating mitochondrial damage/dysfunction and boosting mitophagy.

A long-standing proposition that mitochondrial damage is solely a trigger of inflammasome activation [6, 7] is now being challenged by our finding that NLRP3 inflammasome activation magnifies mitochondrial damage. In support of our idea, a couple of studies suggested that NLRP3 inflammasome activation was linked to mitochondrial damage [29, 42]. Thus, we propose that there exists a vicious cycle between mitochondrial dysfunction and NLRP3 inflammasome activation. Importantly, we showed that VX765 could potently break this vicious loop.

In conclusion, our work revealed the novel role and mechanism for VX765 in antagonizing NLRP3 inflammasome assembly. Our study provides significant insights into the mechanisms for NLRP3 inflammasome activation in driving atherosclerosis. Inhibition of caspase 1 by VX765 paves a new avenue to combat ASCVD.

## Supplementary information

Original western blots

Reproducibility Checklist

Author Contribution Statement

SUPPLEMENTARY FIGURE LEGENDS

Supplementary Fig. 1 The effect of VX765 itself on mitochondrial damage and pyroptosis.

Supplementary Fig. 2 Activated NLRP3 inflammasome amplifies mitochondrial damage and VX765 inhibits cell death.

Supplementary Fig. 3 The effect of VX765 on ABCA1 expression in ApoE-/- BMDMs.

Supplementary Fig. 4 The expression of interleukin-1β and its regulation by VX765 in smooth muscle cells in atherosclerotic plaques.

Supplementary Fig. 5 The expression of NLRP3 in the aortic sinuses of ApoE-/- and Nlrp3-/-;ApoE-/- mice.

## Data Availability

Data supporting the findings of this study are available from the corresponding authors on reasonable request.
