# Supplementary figures and images for: Novel role for caspase 1 inhibitor VX765 in suppressing NLRP3 inflammasome assembly and atherosclerosis via promoting mitophagy and efferocytosis
Source: Cell Death Dis. 2022 May 31;13(5):512. doi: 10.1038/s41419-022-04966-8 (PMC9156694; doi:10.1038/s41419-022-04966-8)

# **Supplementary Data 2**

**(Uncropped blots)**

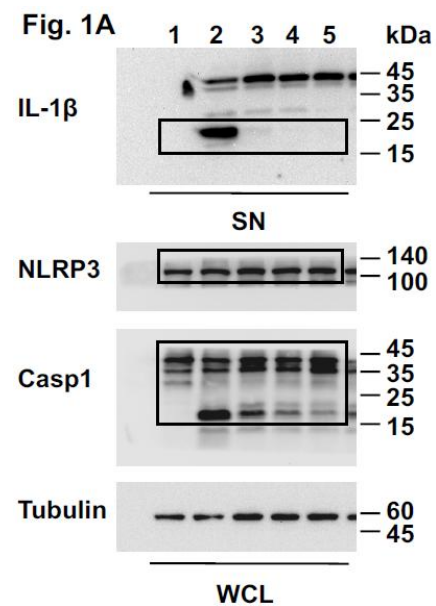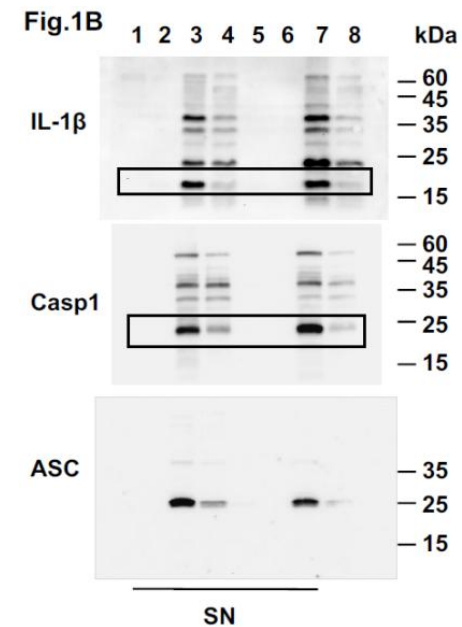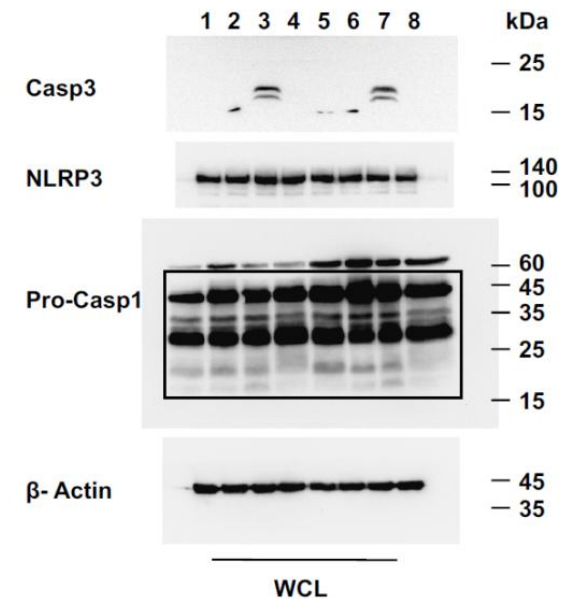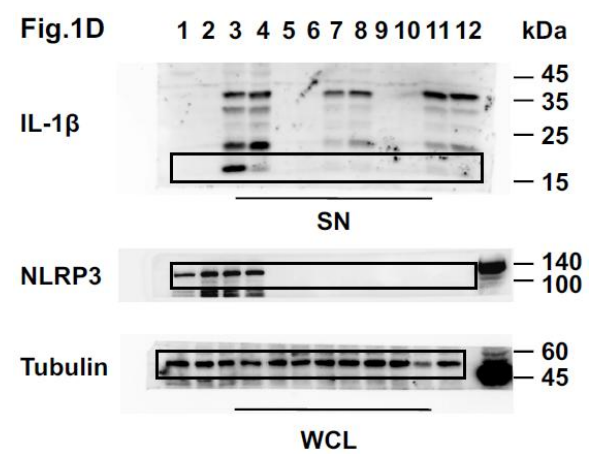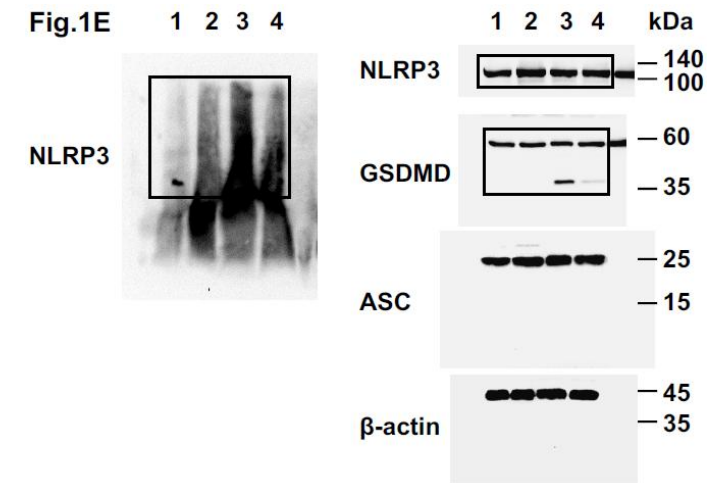

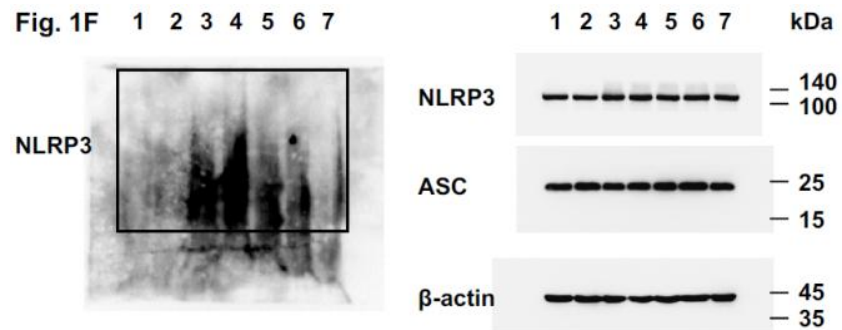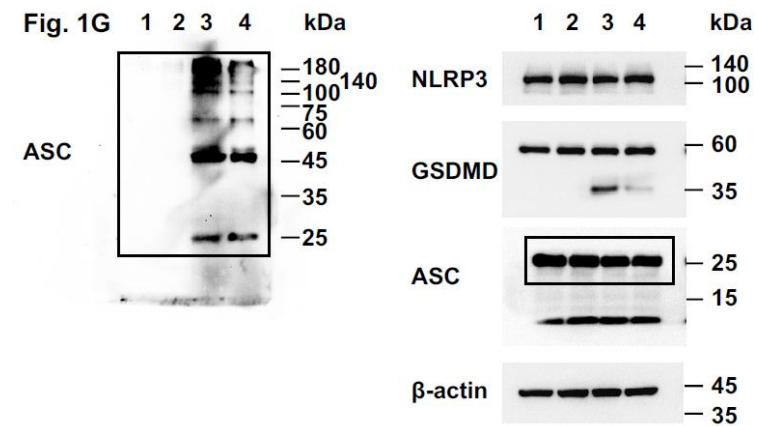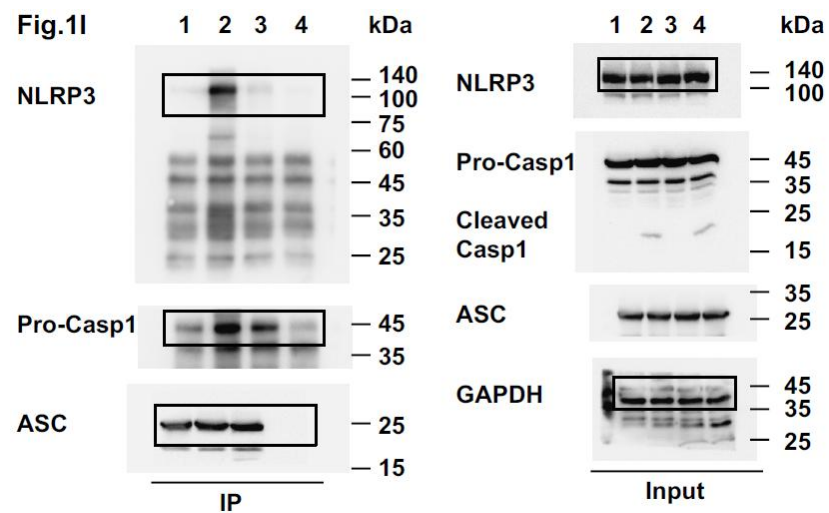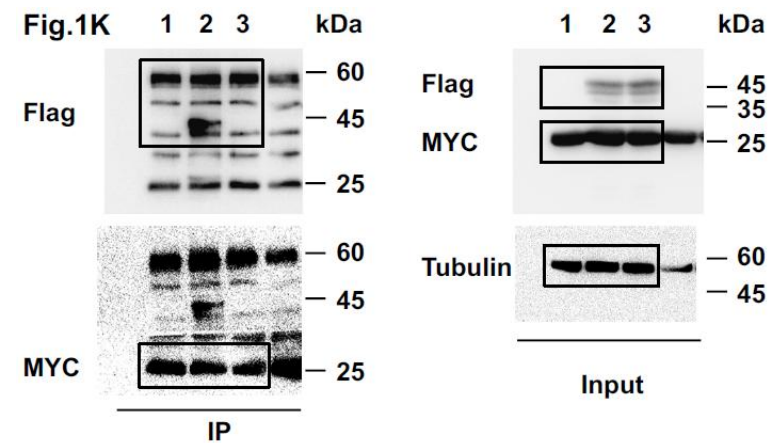

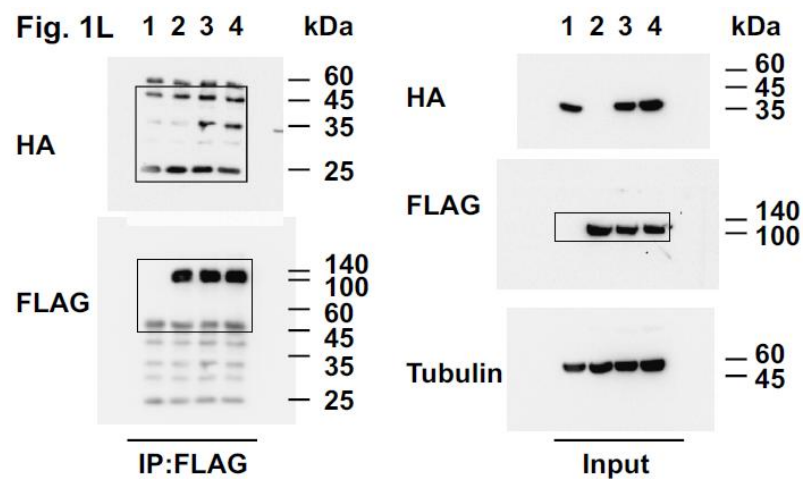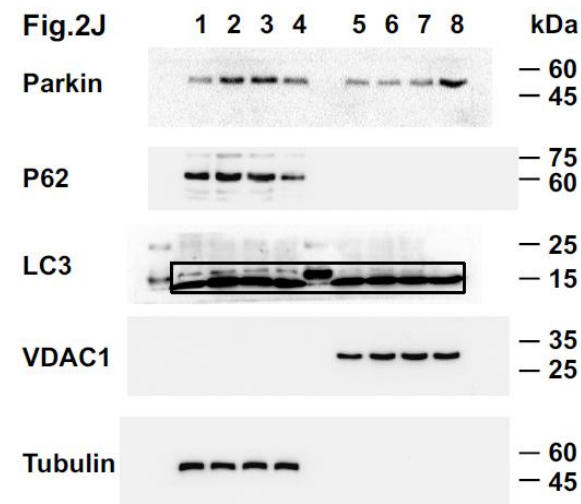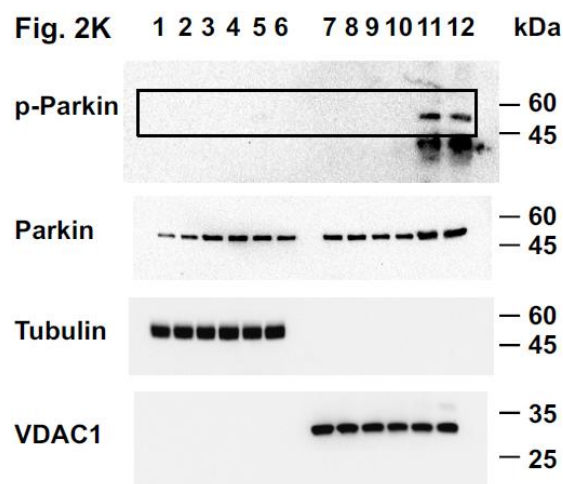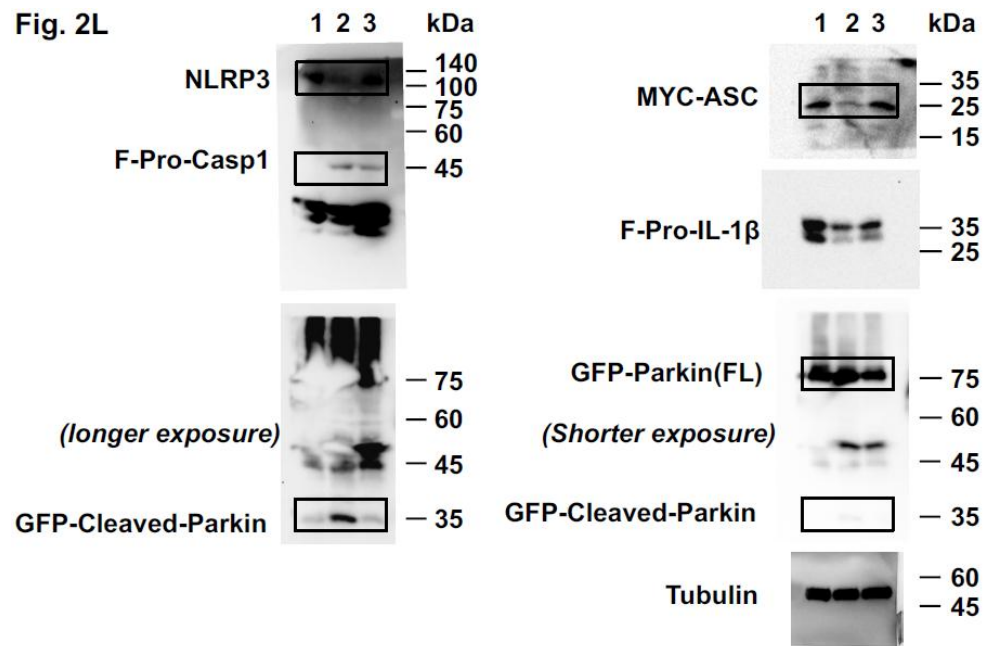

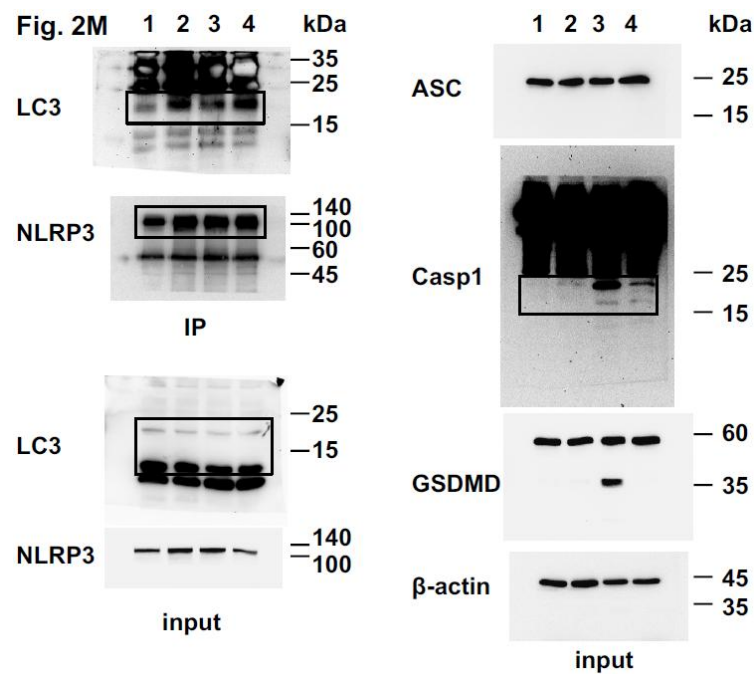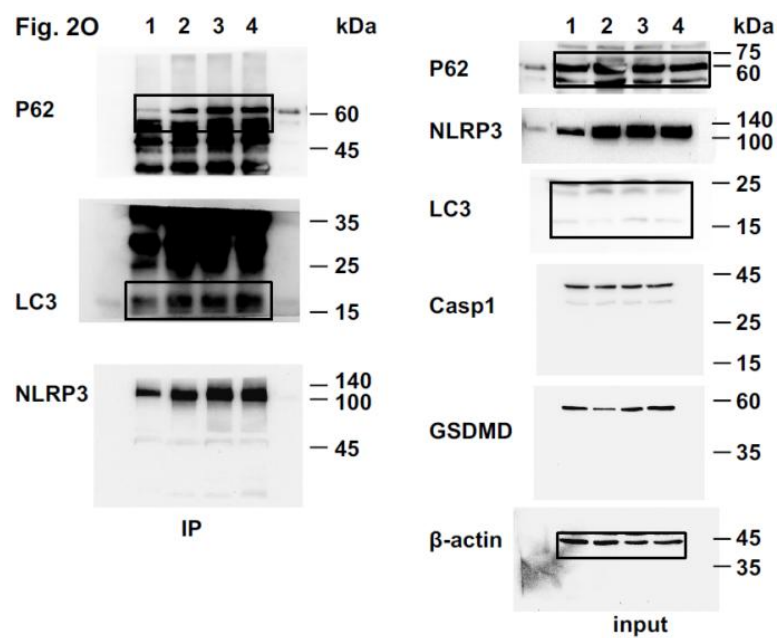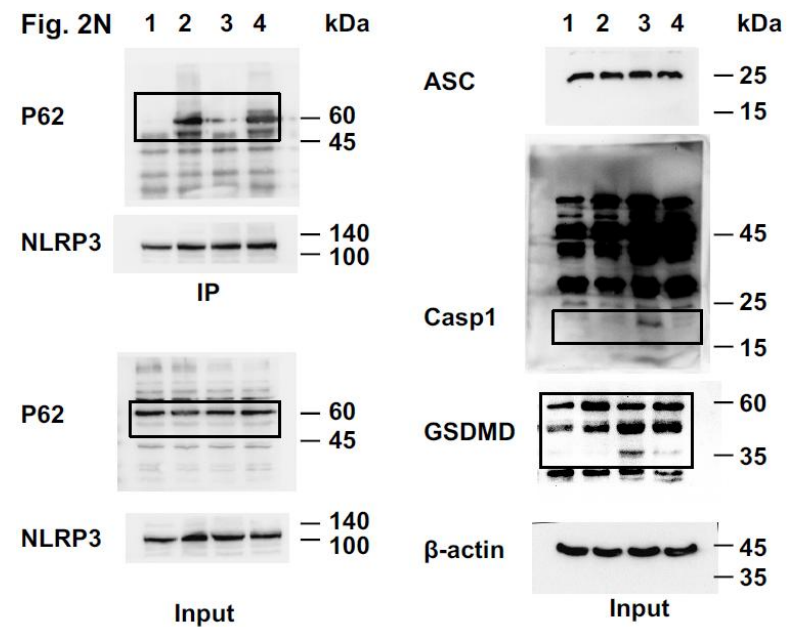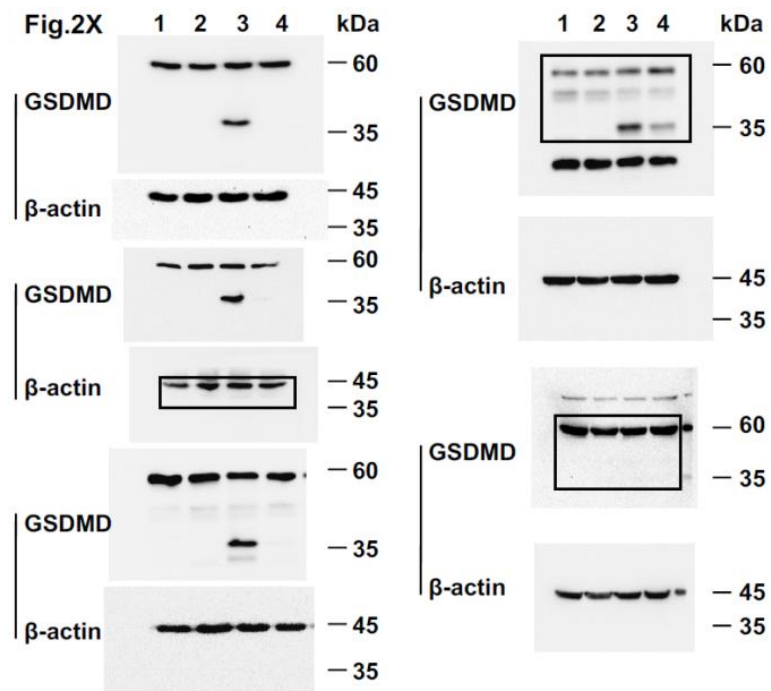

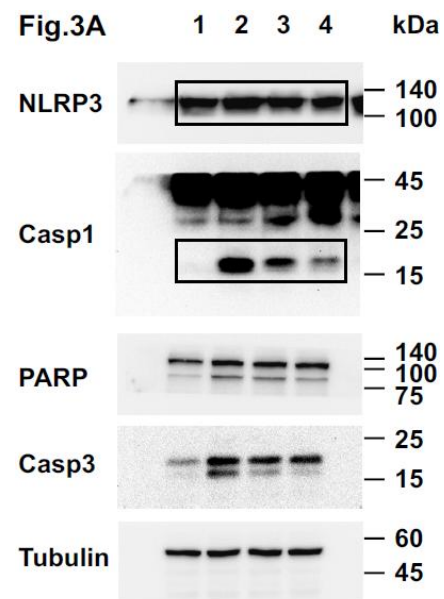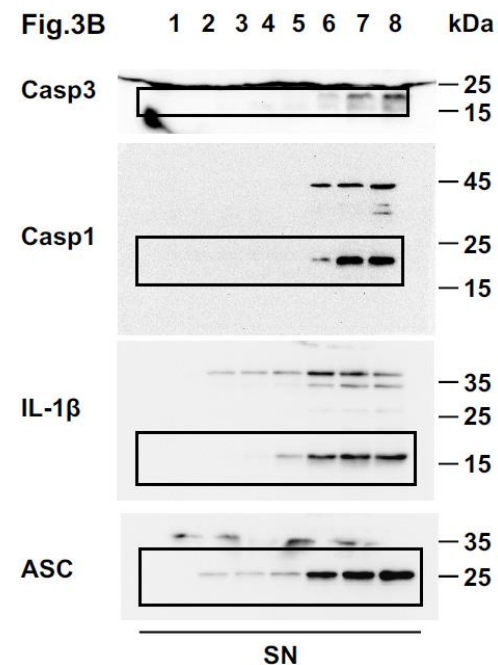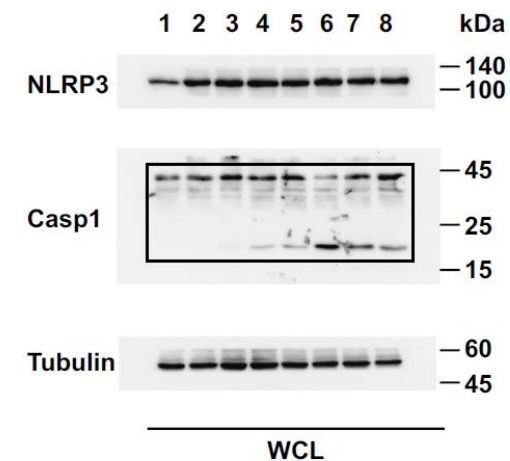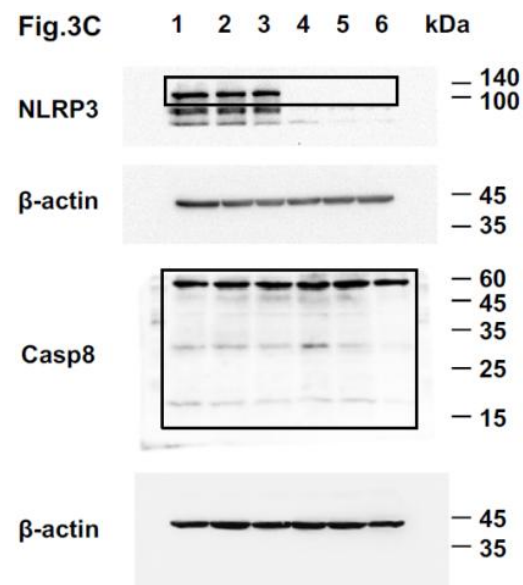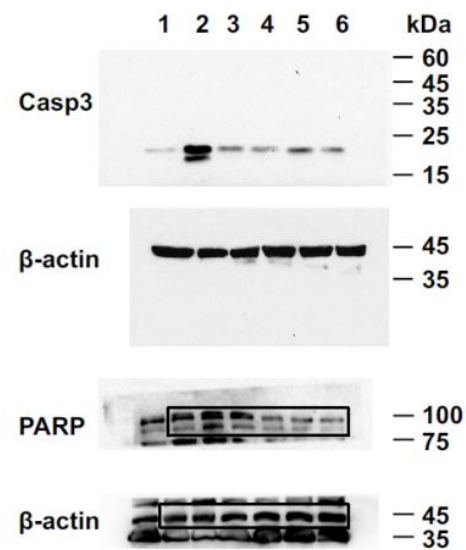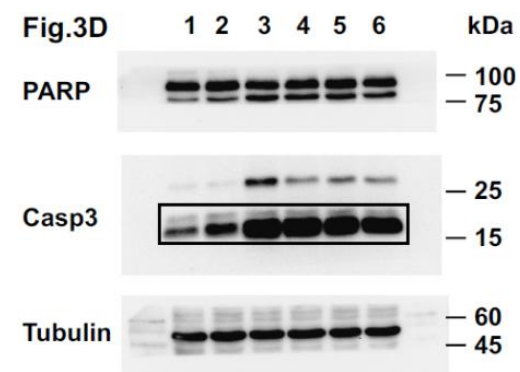

Supplement: Supplementary file 1 — Original western blots [file 41419_2022_4966_MOESM1_ESM.pdf]

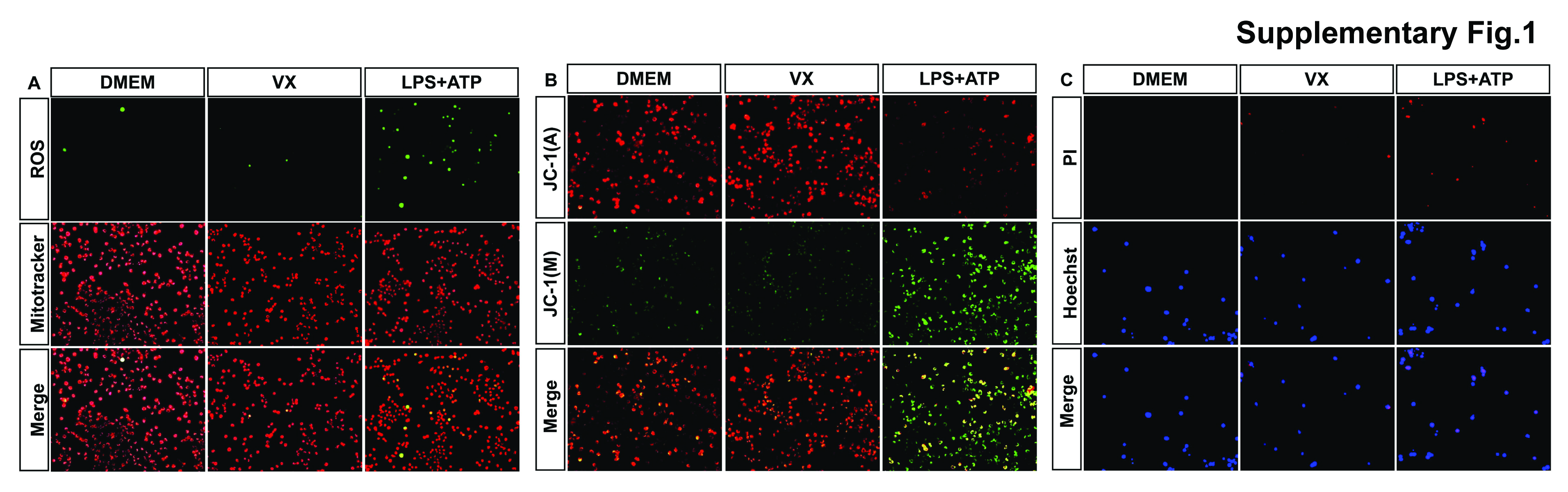

Supplement: Supplementary file 5 — Supplementary Fig. 1 The effect of VX765 itself on mitochondrial damage and pyroptosis. [file 41419_2022_4966_MOESM5_ESM.tif]

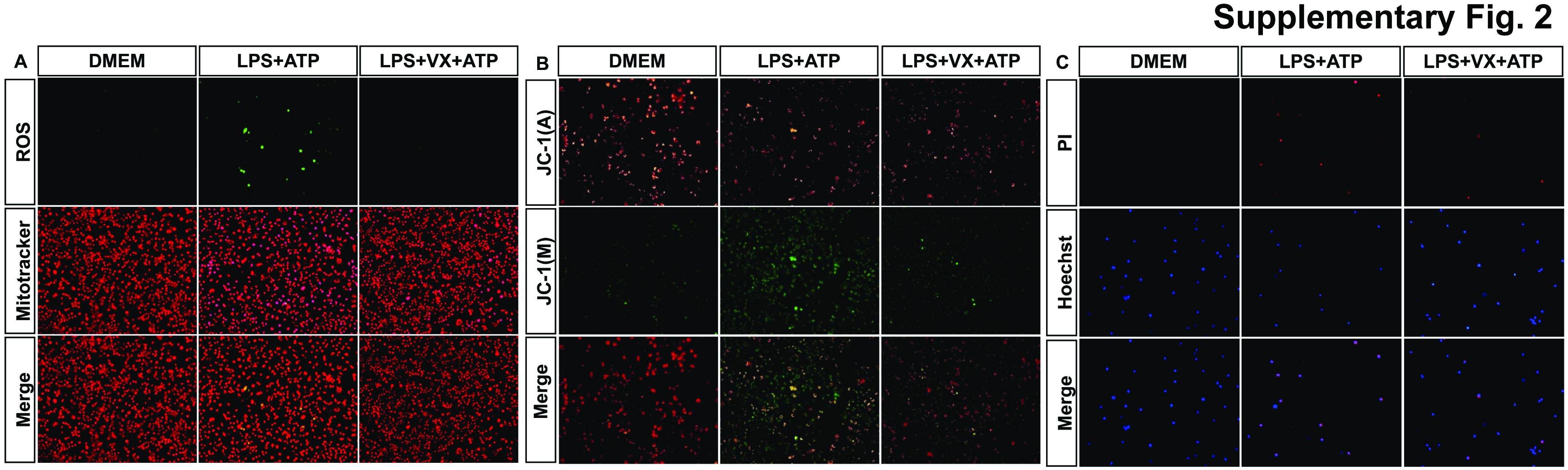

Supplement: Supplementary file 6 — Supplementary Fig. 2 Activated NLRP3 inflammasome amplifies mitochondrial damage and VX765 inhibits cell death. [file 41419_2022_4966_MOESM6_ESM.tif]

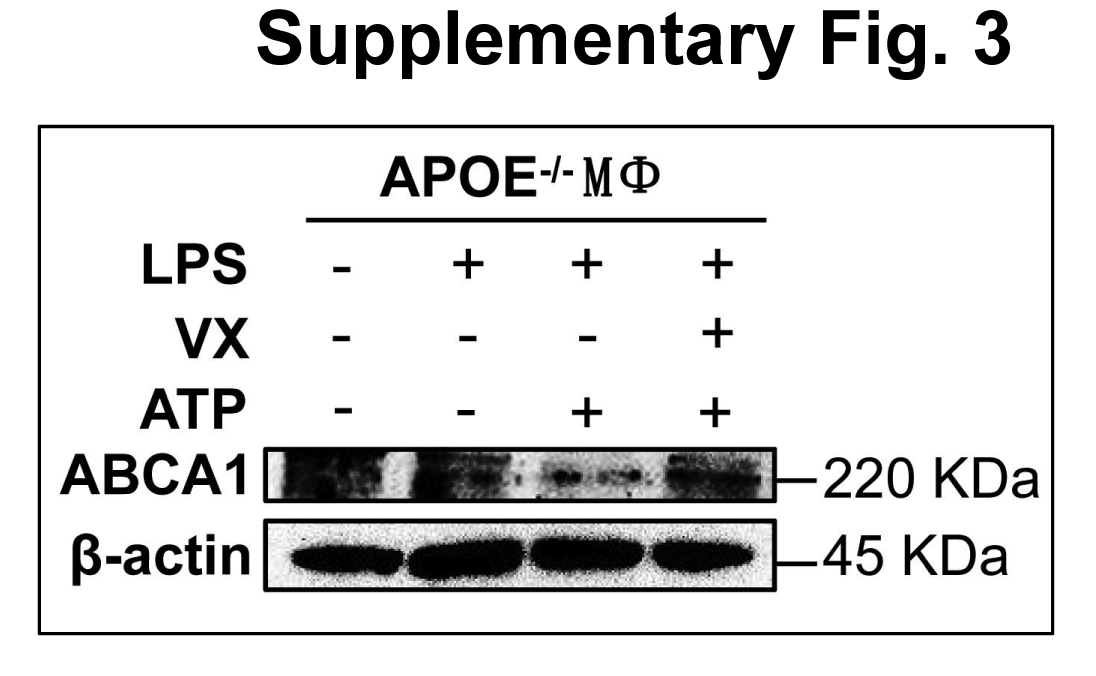

Supplement: Supplementary file 7 — Supplementary Fig. 3 The effect of VX765 on ABCA1 expression in ApoE-/- BMDMs. [file 41419_2022_4966_MOESM7_ESM.tif]

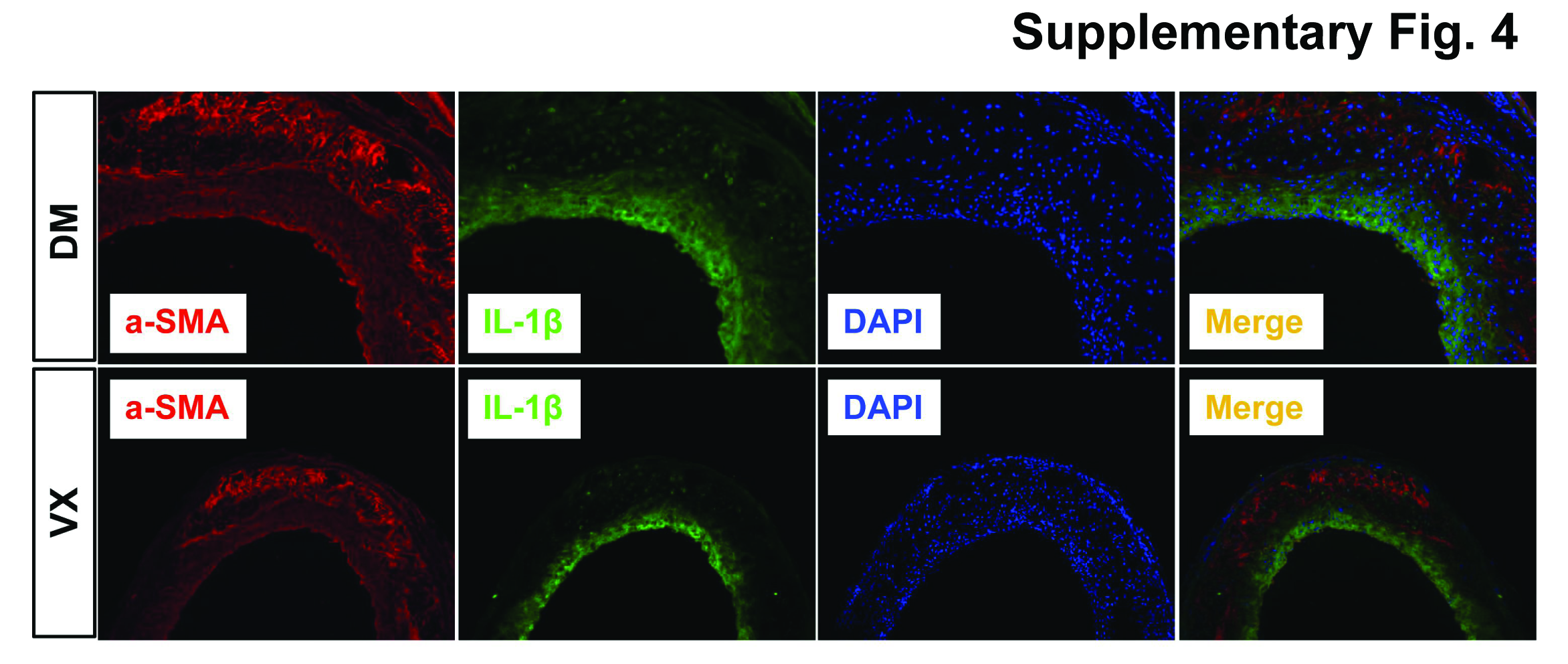

Supplement: Supplementary file 8 — Supplementary Fig. 4 The expression of interleukin-1β and its regulation by VX765 in smooth muscle cells in atherosclerotic plaques. [file 41419_2022_4966_MOESM8_ESM.tif]

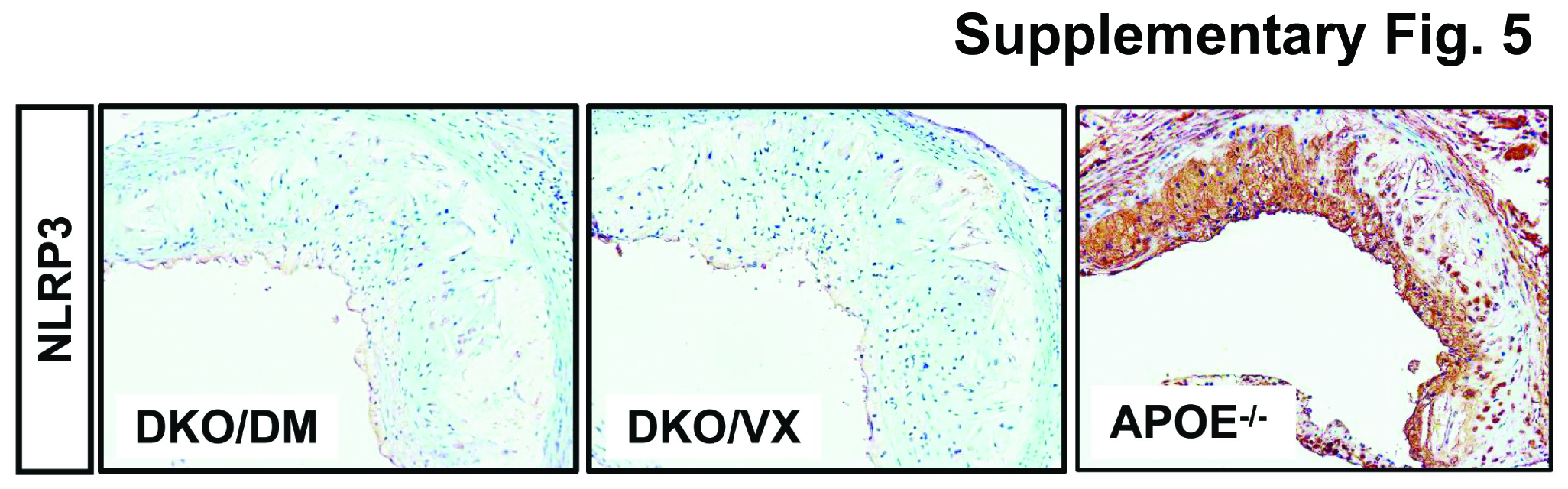

Supplement: Supplementary file 9 — Supplementary Fig. 5 The expression of NLRP3 in the aortic sinuses of ApoE-/- and Nlrp3-/-;ApoE-/- mice. [file 41419_2022_4966_MOESM9_ESM.tif]
