# Supplementary material for: Novel role for caspase 1 inhibitor VX765 in suppressing NLRP3 inflammasome assembly and atherosclerosis via promoting mitophagy and efferocytosis
Source: Cell Death Dis. 2022 May 31;13(5):512. doi: 10.1038/s41419-022-04966-8 (PMC9156694; doi:10.1038/s41419-022-04966-8)
Supplement: Supplementary file 3 — Author Contribution Statement [file 41419_2022_4966_MOESM3_ESM.docx]

**Authors’ Contribution Statement**

**Yao Liu, Lei Xu,** **Liguo Tan,** **HongXian Song,** and **Ying Jin** acquired data and elucidated the role and mechanism for VX765 in atherosclerosis in the mouse models;

**Yao Liu,** **Lei Xu,** **Yulian Xiong, Ying Jin,** and **Jian Fu** acquired data and uncovered the role of VX765 in regulating atherosclerosis-associated properties of macrophages;

**Yao Liu, Ke Ding,** **Yulian Xiong, Shuang Zheng,** and **Ying Jin** acquired data and established the role of VX765 in regulating NLRP3 inflammasome activation;

**Jie Xu, Yazhi Peng, Lei Xu,** **Yulian Xiong,** and **Jian Fu** acquired data and clarified the role of VX765 on NLRP3 inflammasmome assembly;

**Lei Xu,** **Jie Xu,** **Yulian Xiong,** **Ying Jin,** and **Jian Fu** acquired data and established the role of VX765 in regulating mitochondrial dysfunction;

**Jie Xu, Lei Xu, Yazhi Peng, Yulian Xiong,** **Nan Yang,** and **Jian Fu** acquired data and determined the impact of VX765 on mitophagy;

**Yao Liu, Zemei Zhang**, **Shuang Zheng**, **Nan Yang**, **Lin Li,** and **Jian Fu** acquired data and explored the effect of VX765 on caspase 3 activation and apoptosis;

**Ying Jin** and **Jian Fu** conceived and designed the experiments;

**Ying Jin, Yao Liu,** **Lei Xu,** and **Jian Fu** analyzed the data and played an important role in interpreting the results;

**Ying Jin** and **Jian Fu** wrote the manuscript;

**Ying Jin** and **Jian Fu** secured funding.
