# Supplementary material for: Novel role for caspase 1 inhibitor VX765 in suppressing NLRP3 inflammasome assembly and atherosclerosis via promoting mitophagy and efferocytosis
Source: Cell Death Dis. 2022 May 31;13(5):512. doi: 10.1038/s41419-022-04966-8 (PMC9156694; doi:10.1038/s41419-022-04966-8)
Supplement: Supplementary file 4 — SUPPLEMENTARY FIGURE LEGENDS [file 41419_2022_4966_MOESM4_ESM.docx]

**SUPPLEMENTARY FIGURE LEGENDS**

**Supplementary Fig. 1 The effect of VX765 itself on mitochondrial damage and pyroptosis.**

J774A.1 cells were treated as indicated. The cells were then stained with DCFH-DA and Mitotracker (**A**) , JC-1 (**B**), and PI/Hoechst 33342 (**C**). ROS reactive oxygen species; VX VX765; LPS Lipopolysaccharides; PI Propidium iodide.

**Supplementary Fig. 2 Activated NLRP3 inflammasome amplifies mitochondrial damage and VX765 inhibits cell death.**

Immortalized BMDMs were treated without or with LPS plus ATP ± VX765. The cells were then stained with DCFH-DA and Mitotracker (**A**), JC-1 (**B**) and PI/Hoechst 33342 (**C**). ROS reactive oxygen species; VX VX765; LPS Lipopolysaccharides; PI Propidium iodide.

**Supplementary Fig. 3 The effect of VX765 on ABCA1 expression in *ApoE^-/-^* BMDMs.**

ApoE^-/-^ BMDMs were treated with LPS and ATP ± VX765 (28 μg/mL). Cell lysates were analyzed by immunoblotting. Mφ macrophages; LPS Lipopolysaccharides.

**Supplementary Fig. 4 The expression of interleukin-1β and its regulation by VX765 in smooth muscle cells in atherosclerotic plaques.**

The aortic sinuses in *Ldlr^-/-^* mice treated with VX765 or vehicle were analyzed by immunofluorescent staining with the indicated antibodies. α-SMA α-smooth muscle cell actin; IL-1β interleukin-1β.

**Supplementary Fig. 5 The expression of NLRP3 in the aortic sinuses of *ApoE^-/-^* and *Nlrp3^-/-^;ApoE^-/-^* mice.**

The expression of NLRP3 in the aortic sinuses was determined by immunohistochemical staining.
